# OpenSimRoot: widening the scope and application of root architectural models

**DOI:** 10.1111/nph.14641

**Published:** 2017-06-27

**Authors:** Johannes A. Postma, Christian Kuppe, Markus R. Owen, Nathan Mellor, Marcus Griffiths, Malcolm J. Bennett, Jonathan P. Lynch, Michelle Watt

**Affiliations:** ^1^ Plant Sciences Institute of Bio and Geosciences 2 Forschungszentrum Jülich Wilhelm‐Johnen Straße 52425 Jülich Germany; ^2^ Centre for Mathematical Medicine and Biology School of Mathematical Sciences University of Nottingham Nottingham NG7 2RD UK; ^3^ Centre for Plant Integrative Biology University of Nottingham Nottingham LE12 5RD UK; ^4^ Plant & Crop Sciences Division School of Biosciences University of Nottingham Nottingham LE12 5RD UK; ^5^ Department of Plant Science Pennsylvania State University 102 Tyson Building University Park PA 16802 USA

**Keywords:** functional–structural plant model, model‐driven phenotyping, OpenSimRoot, plant nutrition, root architectural traits, root system architecture, simulation

## Abstract

- OpenSimRoot is an open‐source, functional–structural plant model and mathematical description of root growth and function. We describe OpenSimRoot and its functionality to broaden the benefits of root modeling to the plant science community.
- OpenSimRoot is an extended version of simroot, established to simulate root system architecture, nutrient acquisition and plant growth. OpenSimRoot has a plugin, modular infrastructure, coupling single plant and crop stands to soil nutrient and water transport models. It estimates the value of root traits for water and nutrient acquisition in environments and plant species.
- The flexible OpenSimRoot design allows upscaling from root anatomy to plant community to estimate the following: resource costs of developmental and anatomical traits; trait synergisms; and (interspecies) root competition. OpenSimRoot can model three‐dimensional images from magnetic resonance imaging (MRI) and X‐ray computed tomography (CT) of roots in soil. New modules include: soil water‐dependent water uptake and xylem flow; tiller formation; evapotranspiration; simultaneous simulation of mobile solutes; mesh refinement; and root growth plasticity.
- OpenSimRoot integrates plant phenotypic data with environmental metadata to support experimental designs and to gain a mechanistic understanding at system scales.

## Introduction

Functional–structural plant models combine a representation of three‐dimensional (3D) plant structure with physiological functions to advance plant science and its applications (Vos *et al*., 2010; Dunbabin *et al*., 2013). Those that incorporate below‐ground root parameters (Dunbabin *et al*., 2002; Pagès *et al*., 2004; Pierret *et al*., 2007; Wu *et al*., 2007; Javaux *et al*., 2008; Leitner *et al*., 2010; Lobet *et al*., 2014; Gérard *et al*., 2017) require significant time and expertise in biological, mathematical, computational and digital image analyses, and therefore their development benefits greatly from an open and global setting. simroot is one of the most feature‐rich and highly cited functional–structural root architectural models. However, the last full description dates back 20 yr (Lynch *et al*., 1997), and subsequent papers have reported applications of the model, with successive changes embedded in methods sections (Postma & Lynch, 2011a,b; Dathe *et al*., 2013). Here, we describe fully a new, open‐source version, branded OpenSimRoot, which is freely available for download (http://rootmodels.gitlab.io/OpenSimRoot). New features in this version allow the simulation of more growth scenarios and crops, and its application has been widened to support emerging root phenotyping technologies.

simroot was originally designed to reconstruct root system architecture (RSA, see Table 1) from empirical data, such as growth rates, angles and branching frequencies of different root classes. A post‐simulation analysis of root geometry, nutrient uptake and carbon (C) costs enabled the comparison of different RSAs with respect to their efficiency in taking up phosphorus (P) relative to C costs (Nielsen *et al*., 1994, 1997; Lynch & Beebe, 1995; Lynch *et al*., 1997; Ge *et al*., 2000; Rubio *et al*., 2001; Walk *et al*., 2004, 2006). Later versions coupled physiological mechanisms, such as root respiration, nutrient uptake, canopy photosynthesis and RSA, to simulate how the root phenotype dynamically interacts with the soil environment, and how this interaction influences the acquisition of soil resources and, consequently, plant growth (Postma & Lynch, 2011a,b, 2012; Dathe *et al*., 2013, 2016; Postma *et al*., 2014a; York *et al*., 2016). The initial focus was on P capture (Lynch & Beebe, 1995; Ge *et al*., 2000; Ma *et al*., 2001; Postma & Lynch, 2011b), which was later expanded to include C (photosynthesis), nitrogen (N), potassium (K) and water (Postma *et al*., 2008; Postma & Lynch, 2011a; Dathe *et al*., 2013). Microeconomic theory, in which resource acquisition is compared with resource investment costs, has guided the interpretation of results (Lynch, 2007; Postma *et al*., 2014b). Although simroot was designed as a heuristic model, i.e. a tool for exploring the implications of existing knowledge and gaps in that knowledge, it proved surprisingly accurate for the prediction of fitness outcomes of root phenotypes (Chen *et al*., 2011; Saengwilai *et al*., 2014; Zhan *et al*., 2015).

**Table 1:** Definition of terms

Term	Definition
State variable	A quantity that has a unit and may depend on time and/or space
Minimodel	An object that encapsulates a state variable and is of a type derived from simulabase (Supporting Information Notes S3). Minimodels place state variables in a context, give them a lifetime, a name, a unit and provide a general application programming interface (API) for coupling of minimodels
Module	A set of minimodels that together form a major component, such as the carbon, nutrient or water modules
Plugin	A class which adds functionality to the model without changing the main code (e.g. see Notes S4). Plugins can be of derived type ObjectGenerator, DerivativeBase or IntegrationBase
ObjectGenerator	Plugin which instantiates new minimodels
DerivativeBase	Base classes for plugins that add new computational ability and/or new dependences among minimodels
IntegrationBase	Base classes for plugins that add new integration procedures
CLI	Command line interface, as opposed to a graphical user interface
Root segment, root, root system, root system architecture (RSA)	Root segment is a short piece of root that can be represented by two coordinates. Root is a single root axis, without branches, unless it stands in contrast with shoot, whereby it represents the whole root system (as in ‘root to shoot ratios’). Root system is a system of connected roots. Root system architecture is the spatio‐temporal arrangement of the root system (Lynch, 1995), and is characterized by RSA traits, such as branching frequencies or root gravitropism. RSA is often described by its geometric attributes, such as depth, width, specific root length, etc.

simroot is one of several root models that have been developed. Dunbabin *et al*. (2013) present an exhaustive review of all root models to date and their capabilities. To our knowledge, OpenSimRoot is currently the only plant root model that is openly version controlled (GIT) and GPLv3 licensed, allowing community‐driven development. We envisage that OpenSimRoot will be used and expanded by both modelers and non‐modelers to simulate RSA and nutrient and water uptake in an ever‐widening scenario for species, environments and crop management practices to advance root‐based opportunities to increase resource‐efficient agricultural productivity. A design goal of OpenSimRoot is a flexible model structure that can be controlled by the user rather than the programmer. This means that, through a plugin infrastructure, the user can directly vary components of the model and compare the results. Model behavior can be studied further through sensitivity analysis, which has been a major focus in past publications.

In this article, we initially provide a short description of the design of the OpenSimRoot model and definitions, and then present the major submodels in OpenSimRoot which simulate RSA, the shoot, C, water and nutrient acquisition and utilization, root growth plasticity and geometric descriptors. After this model description, we discuss model implementation, which is designed for flexibility, extensibility, transparency and robust numerics. We conclude with several examples of OpenSimRoot usage.

## Materials and Methods

Compared with other root models, OpenSimRoot has a unique design which centers on the coupling of various minimodels (for definitions, see Table 1). In line with object‐oriented programming, the distinction between parameter and algorithm has been removed by encapsulating both within classes which share a common interface for coupling and data exchange.

### OpenSimRoot design

**Figure 1:**
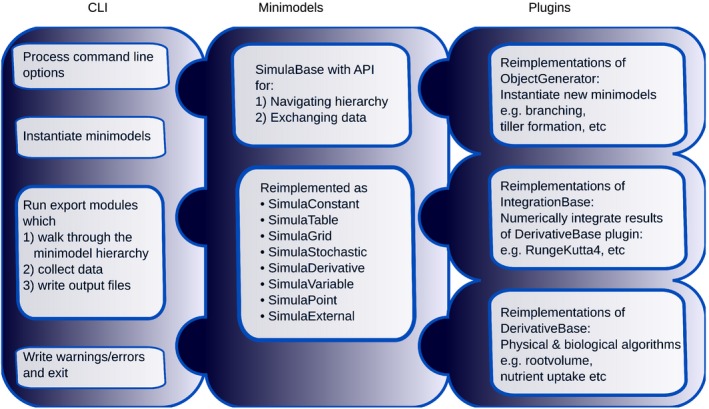
Schematic representation of the OpenSimRoot code. The code encompasses three major components: the command line interface (CLI), different types of minimodels and a library of plugins. The class hierarchy for each component is given in Supporting Information Notes S3.

OpenSimRoot contains a command line interface (CLI), a simulation engine, a plugin library and classes responsible for the reading and writing of data (Fig. 1; Supporting Information Notes S1–S4). The simulation engine implements an application programming interface (API) through which different modules can request information (see Notes S1). The plugin infrastructure allows developers to implement new modules with limited knowledge about the rest of the code. Each plugin establishes dependences between minimodels through the API and requests data from other minimodels in order to compute the necessary information. At the start of execution, the import module reads an XML file (see below) and, based on that file, constructs a tree of minimodels. According to the specification in the XML, the minimodels load (instantiate) appropriate algorithms from a registry which lists all available plugins (Notes S3). The plugin infrastructure not only allows the user to implement new processes, but also to implement alternative algorithms and to compare model results. The behavior of the modules described below is thus not fixed, but can be adapted to hypotheses. Simulation is driven on the basis of data requests that originate from the user's request for output. On instantiation of the object tree, the modules that write output start to request information in order to write the output files. The CLI has a small number of options (listed with ‐h) with the most important being the input file name. Runs are non‐interactive, such that many runs with different parameter combinations can be fully automated on a computational cluster. This capability is important when large numbers of simulations are required, for example when exploring parameter sensitivity or when processing real root structures (see for examples the Results section) from large numbers of plants.

### Description of the various modules

#### Root growth and RSA

The root system is represented by vertices and edges in OpenSimRoot. Every root tip has its own vertex with dynamic coordinates, and all other vertices have stationary coordinates that are placed behind the root tip as it extends. The final discretization of the root system can be coarser than the frequency of each growth point's directional change. A fine‐scale discretization request can automatically reduce the integration time step. In the case of a coarse discretization, the length of a root segment is not the linear distance between two vertices, but the true distance that the root grows, based on the growth rate at that given time. We thus ‘simplify’ the growth trajectory for computational reasons, without losing the true root length.

**Figure 2:**
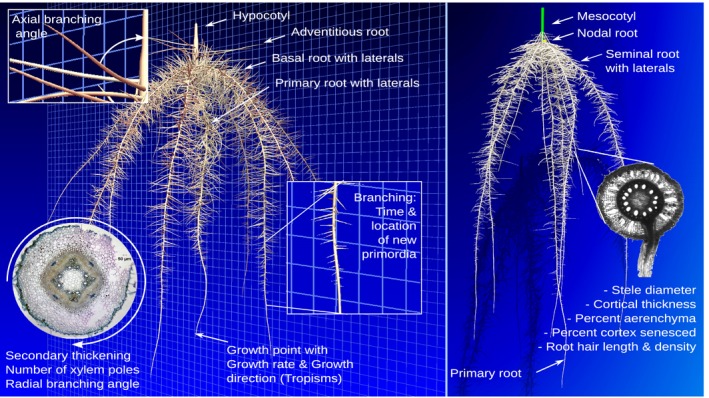
Simulated root system of bean (left) and maize (right) as rendered with paraview. Root systems are made up of different root classes, each with their own root diameter, branching rules, growth direction and growth rates. Root cross‐sections are not simulated, but illustrate root segment traits that are represented in OpenSimRoot.

To grow a root system, we need to know: (1) when and where the root tips (primordia) are created; (2) how fast the root tips grow; and (3) in what direction. To start, we assume that, at a minimum, one primary root and a hypocotyl are present in the seed embryo. The term hypocotyl is used here freely to include any shoot axiles (stems) that are the origins of adventitious roots, whether simulating dicotyledons or monocotyledons. Branch roots and their own branch roots (classed according to order) are assumed to emerge from the primary root, based on rules that control the timing and placing of the branches. Adventitious roots (crown or nodal roots in grasses) can branch from the stem according to different schemes, the simplest being defined by a starting time and position of a single whorl of roots. The formation of branch roots from these axiles is typically based on branching frequencies, which can be expressed in time, space or both, where the missing information is computed on the basis of the growth rate of the parent root. Roots can branch from either phloem or xylem poles, depending on species (Casimiro *et al*., 2003). The number of poles determines the number of positions of the radial branching angle, and the axial branching angle (angle between the parent root and branch) is given in the parameter section (for a detailed explanation, see Lynch *et al*. (1997) and Fig. 2).

The elongation rates of individual roots are predefined in the parameter space, but may be scaled according to a ‘root vigor’ scaling factor, for example, drawn from a lognormal distribution of elongation rates (scaled to unity), thus creating variation in length. The vigor factor can also scale the root‐specific root diameter to allow an allometric relation between elongation rate and root diameter expansion within a root class (Pagès, 2000; Wu *et al*., 2016). The initial root diameter is otherwise a root class‐specific input parameter.

Although the initial growth direction is set by specified radial and axial branching angles (Fig. 2), the direction can be changed with a tropism vector. The tropism vector is the sum of several vectors representing gravitropism, random impedance and nutrient tropisms, and is added to the normalized growth direction vector to obtain the new direction.

Once the root is growing, its branching rules allow it to branch off new roots of different classes and the whole process is repeated. Although OpenSimRoot currently does not simulate shoot architecture, a simple tiller model is included. Tillers can form their own leaf area and their own root systems. In grasses, tillers produce nodal roots which can form a significant fraction of the total root system, depending on species and environment (Atkinson *et al*., 2014; Sebastian *et al*., 2016). Tiller formation is performed on the basis of a table that indicates the time‐dependent delay until the next tiller is formed. Dicotyledonous roots have secondary growth from cambia which thicken the stele and periderm in the root. Secondary growth is simulated using a time‐dependent radial growth rate scaled to distance along the root.

#### Simulation of shoot growth and related processes

A simple shoot model can be constructed with OpenSimRoot plugins. The shoot model is non‐geometric and represents the shoot by the state variables leaf area and leaf and stem dry weight. The increase in dry weight is based on C allocated to leaves and stems, multiplied by a dry weight to C factor. The increase in leaf area is the increase in leaf dry weight multiplied by the specific leaf area (SLA). Carbon partitioning can be based on predefined time‐dependent values (van Ittersum *et al*., 2003). Carbon partitioning tables are typically established from dry weight measurements and thus, instead of entering C partitioning tables, OpenSimRoot can also compute partitioning directly from dry weight measurements. This predefined growth represents ‘potential’ growth under a well‐watered and fertilized condition, whereas nutrient or C limitations may alter C partitioning (see below). Total C available for plant growth is computed by subtracting the C costs (for example, respiration and root exudates) from the total C fixed in the leaves and/or available from seed or non‐structural C reserves. Carbon costs depend on rates of respiration or C expenditure on exudates or nitrate uptake, and these are integrated over the whole plant or root system. Total C fixation is based on a radiation use efficiency (RUE) model, whereby intercepted light is converted linearly to C fixation. Intercepted light is computed from the leaf area index, assuming that the simulated plant is in a homogeneous canopy of equally spaced and identical plants. Tillers are simulated as new plants with their own leaf area, but sharing resources.

#### Carbon allocation to roots

The root growth module can compute the C for growth for each root segment (edge) using its volumetric increase and a specific root volume (g cm^−3^). Volume increases arise from primary and/or secondary growth, and root segments are assumed to be cylindrical or, in the case of varying diameters, a truncated cone. OpenSimRoot compares available with required C and, if the source strength is greater than the sink strength, stores the C left over into a labile pool. OpenSimRoot thus considers that plant growth may be physiologically, not resource, constrained (Postma *et al*., 2014b). The labile pool is depleted when the sink strength (defined by C needed for potential growth) is greater than the source strength. Once stored C is depleted, growth rates decline. Various rules for C allocation under source‐limiting conditions have been implemented. The most commonly used rule to date prioritizes shoot over roots and, within the root system, secondary growth (root cambial thickening) over elongation, and, within the root classes, elongation of major bearing roots over branch roots. Consequently, when plant growth is C limited, the growth rates of branch roots are more strongly reduced than the growth rates of parent roots. These rules do not have a physiological basis, but rather a pragmatic basis in which source–sink imbalances are seen as errors in the parameterization and estimation of the growth rates, and the assumption is that these errors are more likely in branch root growth than in shoot growth. However, other rules, such as equal scaling of all organs, have been implemented, and can be used if the user assumes that all sinks compete equally for the available C.

Although the inputs of the model are absolute growth rates, allometric scaling, based on the ratio between actual and potential leaf area (not mass), can reduce the attainable growth rate of the canopy and the rate of formation of new root branches. This implies that plants can never fully recover from a stress. However, a recovery rate can be defined which allows the plant to grow, for example, 10% faster when resources permit. Allometric scaling can also be used for the formation of branches. For example, the number of nodal roots per whorl in maize is dependent on the size of the shoot.

#### Hydrology

**Figure 3:**
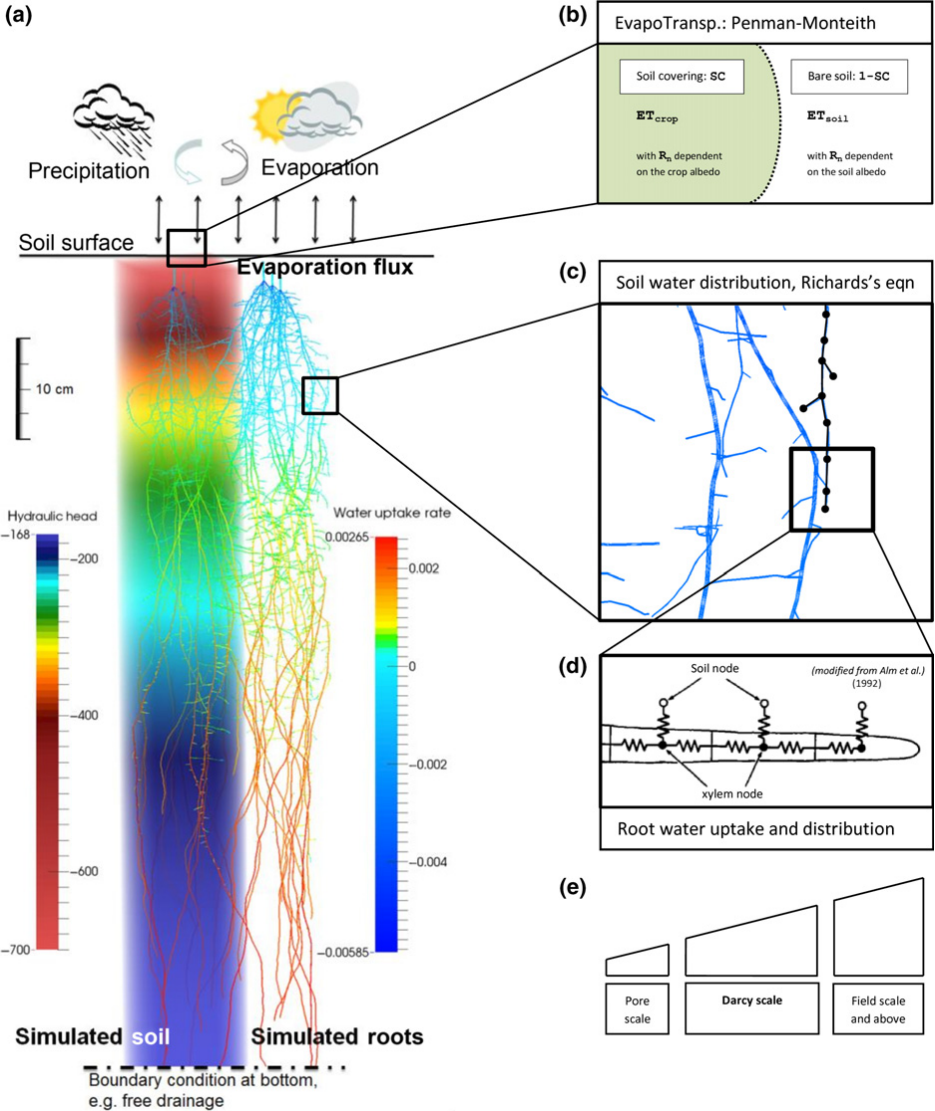
Schematic representation of the coupling of the evapotranspiration, xylem transport and soil water modules. (a) Soil pedon with the hydraulic head indicated in pseudo‐color (left) and three barley root systems (right) taking up water from that column. At the dry top, water uptake is negative, meaning that some hydraulic lift occurs in this scenario. (b) The Penman–Monteith equation for the simulation of transpiration and evaporation. (c) Zoomed version of roots, showing the edges and vertices. (d) Network model for the simulation of water flow through the roots. Modified, with permission, from Alm *et al*. (1992). (e) Water transport in three dimensions in the soil is simulated by solving the Richards equation, which combines Darcy's law with mass conservation, using the finite element method.

OpenSimRoot includes a hydrology module (Fig. 3). The implementation of the hydrology module involves the coupling of three models that simulate the movement of water through the soil and plant and into the atmosphere. OpenSimRoot includes a simplified C++ implementation of the SWMS model which is used to simulate soil water transport in Hydrus (Diamantopoulos *et al*., 2013) and Rswms (Šimunek *et al*., 1995). Water transport through the xylem is simulated using a hydraulic network model (Alm *et al*., 1992; Doussan *et al*., 1998), and evapotranspiration is simulated using the Penman–Monteith equation (Penman, 1948; Monteith, 1964). Small adjustments of these models, to achieve good coupling, are described in Notes S5.

The hydrology module provides 3D water uptake profiles, drives convective nutrient transport and can simulate compensatory water uptake and hydraulic redistribution, which may occur when the top soil dries out, causing nutrient uptake from dry soil domains to be reduced. It currently does not simulate drought‐related growth responses.

#### Nutrients

**Figure 4:**
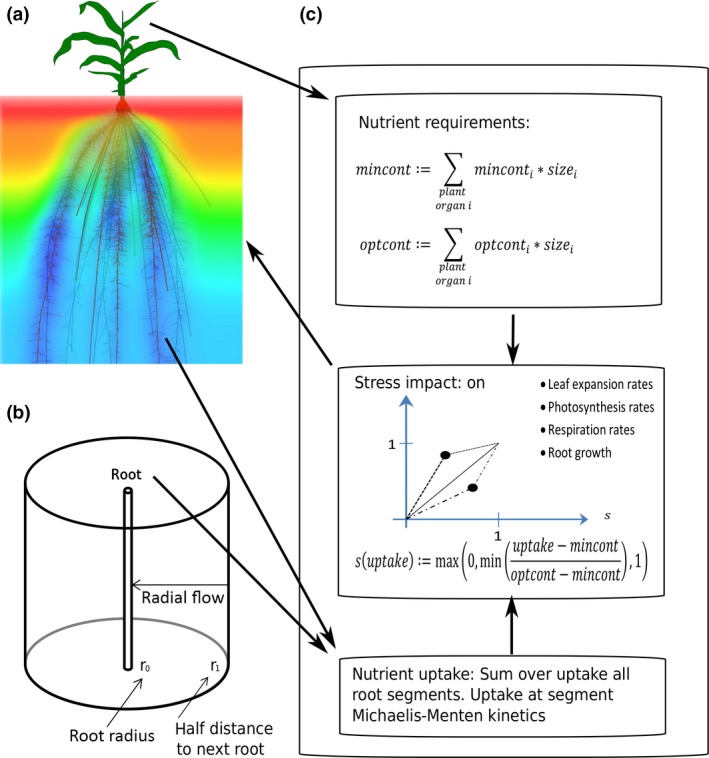
Schematic representation of the nutrient uptake, nutrient requirements and growth regulation modules. (a) Root nutrient uptake coupled to the model for solute transport in the soil. (b) Schematic representation of the radial one‐dimensional Barber–Cushman model used for the simulation of phosphorus (P) uptake. (c) Summary of how the ratio between nutrient requirements and nutrient uptake determines plant physiology and/or growth.

OpenSimRoot has a nutrient module to simulate the simultaneous uptake of solutes, originally implemented to simulate the impact of RSA on nutrient uptake, and to test tradeoffs for the acquisition of nutrients (Postma & Lynch, 2011a; Dathe *et al*., 2013). Postma *et al*. (2014a) showed how the optimal branching density in maize depends on the relative availability of P and N. The module involves three parts: (1) the simulation of plant nutrient requirements; (2) the simulation of nutrient acquisition; and (3) stressors which define how suboptimal plant nutrient concentrations affect physiology or growth (Fig. 4). Nutrients are simulated independently of each other, except that, in step (3), the impact of suboptimal nutrient concentrations on a given state variable is aggregated using a maximum or averaging function. For example, photosynthesis may be affected more strongly by N than P, but P might affect the leaf area expansion rate more strongly (see Dathe *et al*., 2013).

The nutrient requirements of the plant are determined by integrating over the whole plant biomass predefined optimal and minimal nutrient concentrations. The plant acquires nutrients through seed reserves, uptake by the root system and optional N fixation. The uptake of nutrients by the root system is simulated by Michaelis–Menten kinetics, where the movement of nutrients in the soil towards the roots is simulated through convection–dispersion–diffusion equations. OpenSimRoot includes two different implementations for solving these equations: (1) the Barber–Cushman model (Itoh & Barber, 1983), which simulates depletion zones around individual root segments at high resolution, and is suitable for immobile nutrients, such as P; and (2) a reimplementation of the solute model included in Swms3d (Šimunek *et al*., 1995), which couples to the soil water model within the hydrology module (above), simulates the whole soil domain and is suitable for mobile nutrients, such as nitrate. More detailed descriptions of these models are given in Notes S5.

When acquisition falls short of that which is required, plant stress is assumed. Stress impact functions can be defined for components such as leaf expansion rate, photosynthesis rates, respiration rates and root elongation rates or secondary growth. By making the initial response of the shoot stronger than that of the roots, the plant decreases the shoot to root ratios when nutrient deficient (Postma & Lynch, 2011a). OpenSimRoot will move towards a functional equilibrium, although, as a result of the inherent slow nature of growth, and the relatively fast dynamics of other processes, this functional equilibrium might not be reached (Postma & Lynch, 2011b; Postma *et al*., 2014b). The current implementation assumes that, internally, reallocation of nutrients is fast and perfect, such that all organs experience equal stress. This might be true for a nutrient such as N, which typically causes chlorosis everywhere in the shoot, but might not be correct for other nutrients. The importance of the simulation of nutrient redistribution in the plant requires further study, and would require the implementation of a shoot architectural model in which the age and position of individual leaves or canopy strata are simulated.

#### Mineralization and rhizosphere processes

OpenSimRoot implements the Yang and Janssen model for mineralization (Yang & Janssen, 2000). This model assumes the exponential decline of a C pool via aging and a decline in breakdown rate. Based on the C : N ratios of the substrate and microbial biomass, the net mineralization or immobilization of N can be computed. OpenSimRoot assumes that ammonium is readily converted to nitrate, and soil water content and temperature are currently ignored. The implementation of the Yang and Janssen model in OpenSimRoot simulates mineralization for every finite element method node independently, and thus mineralization rates may vary in space. The user can define an N fixation rate as a percentage of the N requirements of the plant. Fixation will not directly reduce N uptake from soil, but will improve plant N status.

Root exudation is not explicitly simulated, but is, instead, described as a root class‐ and time‐dependent C cost. Furthermore, exudation may increase the soluble nutrient concentration in the soil at the cost of the insoluble fraction, and thereby increase nutrient availability locally (Barber–Cushman model only).

#### Root growth plasticity

**Figure 5:**
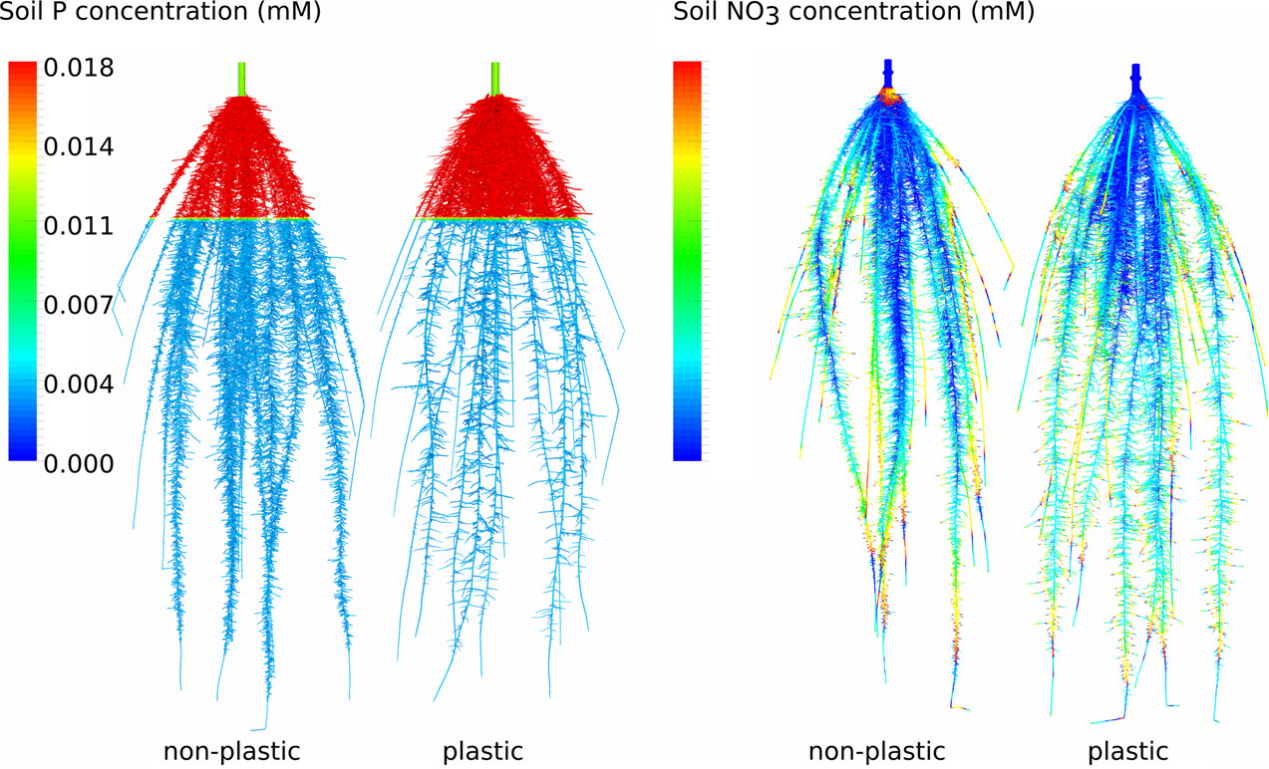
Simulation results for plastic and non‐plastic root systems. Root plasticity is defined as increasing lateral branching density with increasing nutrient availability. Phosphorus (P) availability (left two root systems) is high in the top soil, causing branching density to be high in the top as well. At the same time, the reduced branching density deeper down, as a result of plasticity, allows the plant to grow the individual laterals longer. Pseudo‐colors show the local P availability. Nitrate moves throughout the soil and thereby the plasticity effect is less pronounced and difficult to trace (right two root systems).

OpenSimRoot can define reaction curves to local environmental factors to simulate a localized growth behavior of roots (Fig. 5), often termed ‘plasticity’ (Bradshaw, 1965; Palmer *et al*., 2001). 3D interpolation of available environmental data is used to define values at the root surface. For example, a reaction curve (norm; Pigliucci *et al*., 1996) can describe how gravitropism is scaled according to the local concentration of a nutrient. Similarly, the branching frequency or root elongation rates can be scaled according to a local soil variable. For example, static fields for soil compaction can be defined in three dimensions, using lists of coordinates and associated values in conjunction with a spatial interpolation algorithm. Root elongation can then be defined as a function of local soil compaction.

Currently, only absolute values (scalars) of local environmental variables, such as soil compaction or nutrient concentrations, can be used to simulate plasticity responses. Gradient sensing (i.e. relative values or tensors) of environmental factors may be important for nutrient‐ or hydro‐tropism, or root proliferation responses into enriched patches. However, the biological mechanism for the sensing of gradients is unclear and, currently, no such mechanism has been implemented. OpenSimRoot does, however, include a mechanism to scale the strength of the local plasticity response on the basis of yet another reaction norm which might couple plasticity to whole plant status.

#### Root length distribution and virtual coring

OpenSimRoot can compute several geometric metrics, specifically root length density profiles, virtual coring, root length below D90 for nitrate and overlap of depletion zones. Others, such as explored soil volume, or fractal dimensions, can be computed by the user on the basis of the geometric model output.

#### Root anatomy

Root anatomy is not simulated in 3D explicitly, but OpenSimRoot can represent the stele diameter, thickness of the cortex, degree of cortical senescence, degree of root cortical aerenchyma (RCA) formation, and length, diameter and density of root hairs. These anatomical traits may influence processes at the root segment level, specifically nutrient content, respiration, nutrient uptake and hydraulic conductivity (Fan *et al*., 2003; Hu *et al*., 2014).

### Implementation

OpenSimRoot is written in C++, an object‐oriented programming language. OpenSimRoot couples minimodels, which encapsulate the simulation of a single state variable. State variables are assumed to be associated with time and space and always have a unit. Minimodels are implemented as single C++ classes which inherit from the same base class (named simulabase), such that they all have the same interface (API). This interface allows minimodels to connect to other minimodels and request data. Minimodels may encapsulate a constant, an interpolation table, a random number generator or may make use of helper functions for computation. These helper functions are of the class type IntegrationBase and DerivativeBase, and are registered under their specific names, such that, based on the input, the correct helper function can be instantiated. Helper functions compute a variable and, when associated with an integration function, can be integrated over time. The true functionality of OpenSimRoot is thus dispatched to the helper functions. Through a plugin framework, developers can add new helper functions and thus extend the functionality of the model. Example code for a plugin is given in Notes S4.

Thus, coupling of the state variables is performed through a simple common interface guaranteeing that minimodels are, from a programmer point of view, standalone objects. Computations are quite indifferent as to how dependent variables are computed. This creates high flexibility in the input files, where the state variables can be defined in a variety of ways, i.e. constant, stochastic, interpolation table or based on a plugin (Table 2; Notes S6).

**Table 2:** A simple example of how a simple relative growth rate model can be constructed with OpenSimRoot by coupling two minimodels, one simulating the rate of growth (rgr = 0.1×length) and one that integrates that rate (analytical result would be length = exp(0.1×t))

Declaration of minimodel	Explanation
<SimulaDerivative name=“rootGrowthRate” function=“usePath” unit=“cm/day”>	Declaration of a minimodel named rootGrowthRate which uses the plugin “usePath” to simulate a growth rate with the unit cm d^−1^
<SimulaConstant name=“path” type=“string”> rootGrowth </SimulaConstant>	Declaration of a minimodel named “path” which contains a string of the path to which “rootGrowthRate” needs to be coupled
<SimulaConstant name=“multiplier”> 0.1 < /SimulaConstant>	Declaration of a minimodel named “multiplier” which is a simple constant with which the result of minimodel named by “path” should be multiplied
</SimulaDerivative>	Closing of the declaration of minimodel “rootGrowthRate”, so it is clear that “path” and “multiplier” are owned by it
<SimulaVariable name=“rootGrowth” function=“useName+Rate” integrationFunction=“RungeKutta4” unit=“cm” > 1. </SimulaVariable>	Declaration of a minimodel named rootGrowth, which will use function “useName+Rate” to retrieve data and will integrate that data with the default integration function, RungeKutta4. Start value is 1

The rate calculation is performed using the plugin “usePath” which simply retrieves the length using the declared path and uses the multiplier to calculate the fraction (0.1). The integration is performed by the default integration method, RungeKutta4, which integrates the result computed by the plugin “useName+Rate”. This plugin simply retrieves the values of minimodel “rootGrowthRate”. If the user would like the relative growth rate to be time dependent, the minimodel “multiplier” can be declared as an interpolation table, i.e. <SimulaTable name=“multiplier” …> 0 0.1 10 0.05 </SimulaTable>. Alternatively, stochasticity could be introduced by declaring the multiplier as of class SimulaStochastic. This model is obviously superfluous, and most plugins will implement more complex computations, with more dependencies (see also Supporting Information Notes S6).

One big challenge in coupling independent (mini)models is the implementation of numerical integration when different models have different time steps, and when implicit coupling is desired. In OpenSimRoot, we implemented a general framework for predictor‐corrected methods, by default RungeKutta4, with three components: (1) Interpolation; (2) Prediction; and (3) Dependence tracking. Each minimodel keeps a timetable to interpolate between time steps and return historical information. Different minimodels can run at different time steps, which are, however, synchronized at every globally defined maximum time step. As all data requests loop through the simulabase API, OpenSimRoot tracks forward dependences and predictions, to determine whether to keep the step taken. Interdependent minimodels (for an example graph of dependencies, see Notes S7) update using a predictor corrector method with interpolation to ensure compatibility of time steps. Although the precise order may have some influence on the numerical accuracy or efficiency, there is typically no rational basis on which to prefer any one order of evaluation and it is therefore simply dependent on the order of information requests (typically breadth‐first search, see hierarchical contextualization).

The independent minimodel approach can create a significant computational overhead. However, simulations of RSA are still relatively fast compared with soil, and we regard the ease with which new functionality can be added, with no or little programming effort or knowledge about the rest of the code, as more important than runtime.

The current implementation of OpenSimRoot only depends on the standard C++ libraries (ISO C++11, and a few system libraries for the CLI) and, on our website (http://rootmodels.gitlab.io), we provide directions for compilation and running on Linux, Mac and Windows operating systems.

#### Hierachical contextualization

Many dynamic models are structured along a sequence of events: the ‘time loop’. However, OpenSimRoot represents the plant as a hierarchy of interacting components to allow the main purpose of the understanding of the function of root traits for the whole plant. Minimodels are placed in a simple hierarchy which provides them with context, whereas the object‐oriented paradigm ‘hides’ the internal workings of each component.

#### Dynamic addition of components

OpenSimRoot adds (instantiates) new components during simulation to represent newly grown roots. This contrasts with crop models that represent plant growth by an increase in values of the state variables. Dynamic memory management, connected to an object‐oriented programming paradigm, is a useful programming feature for the addition of new components (Dingkuhn *et al*., 2005). Each minimodel can optionally have a class (inherited from the class ObjectGeneratorBase) attached to it, which, when the children of the minimodel are requested, is run to update the list of children. For example, there are classes that will create new branch roots or will insert new vertices (rootNodes) into the hierarchy. Most of these classes do this by copying templates, which contain all the necessary minimodels that are defined in the input files. An example of an ObjectGenerator plugin is given in Notes S4.

### Input files

OpenSimRoot uses a hierarchical file of parameter values, which not only contains parameter values, but all state variables, and their metadata, such as names and units. Hierarchy provides context, such that parameter lists can be specific for different root classes of different plant species. Input files are implemented in XML, a general language for describing data together with metadata that is also hierarchical, flexible, allows comments, is supported by many software tools and can be rendered in a browser as a more readable document. Notes S6 gives an example of an input file that simulates a simple relative growth model.

**Figure 6:**
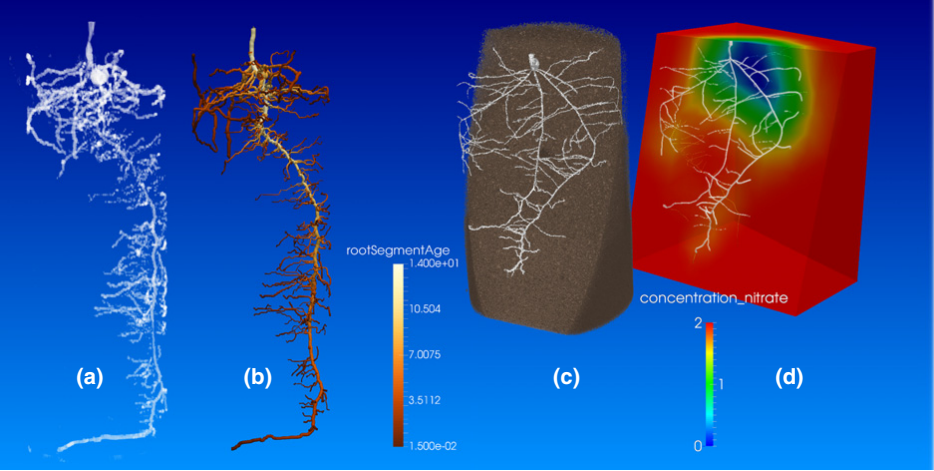
Simulation of imaged root phenotypes. (a) Rendering of a magnetic resonance image of a 2‐wk‐old maize root system and (b) the simulation of that root system by OpenSimRoot. Pseudo‐colors in (b) show the root segment age as estimated on the basis of root topology, linear interpolation and the assumption that the emergence of laterals takes 2 d. (c) Rendering of a segmented X‐ray computed tomography (CT) image of a 10‐d‐old wheat root system. Soil has been sliced to make roots visible. (d) OpenSimRoot simulation of the predicted nitrate depletion zone of the imaged root phenotype in (c). We assumed an initially homogeneous distribution of nitrate within the simulated soil domain. Pseudo‐colors show the nitrate concentration on a plane cut approximately through the center of the root system.

OpenSimRoot allows the user not only to enter initial values, but also arrays of initial time series. In this way, part of the RSA can be predefined, based on measurements (see also examples in the Results section). This approach may be different from most models, but creates the opportunity to use the model as an extension to phenotyping, as partial information derived from phenotypic measurements can be entered directly into the input files (Fiorani & Schurr, 2013; Fig. 6). Parameterizations exist for maize, squash, bean, lupin, *Arabidopsis* and barley, and are now being developed for wheat and rice (Ma *et al*., 2001; Chen *et al*., 2011; Postma & Lynch, 2012). Input files for maize and bean, a predefined root system, a small crop model and other testing scenarios are included in the source code repository (https://gitlab.com/rootmodels/OpenSimRoot).

### Output files

OpenSimRoot includes export modules that can be enabled or disabled to retrieve specified output forms that include tables in text files, 3D models in various VTK (visual tool kit, http://www.vtk.org) formats, 3D raster images and an XML formatted dump of the model in the format of OpenSimRoot's own input files. For example, tables can be further processed with statistical software (like R), VTK files can be opened with 3D data viewers (e.g. paraview, http://www.paraview.org/) and the model dump can be viewed in a web browser (Notes S6).

### License

OpenSimRoot is available under the GPLv3 License (https://www.gnu.org/licenses/gpl-3.0.en.html), which is an open‐source–copyleft license. The license enables the practice of ‘good science’ by making the model transparent and by facilitating contributions from a wider range of expertise in the community. The version controlled code can be accessed at https://gitlab.com/rootmodels/OpenSimRoot.

## Results

### Application examples for OpenSimRoot

simroot has found useful application in several domains, including: (1) geometric analysis of root system form and function; (2) simulation of processes that are very difficult to measure empirically; (3) simulation of dynamic systems; (4) sensitivity analyses; and (5) simulation of hypothetical systems. In addition, a new capability of OpenSimRoot to read in (partially) predefined RSA enables application as an extension to 3D phenotyping techniques, such as X‐ray computed tomography (CT) and magnetic resonance imaging (MRI). Examples of all of these applications are provided below.

### Studies on the function of RSA traits

A primary output of OpenSimRoot is the RSA phenotype emerging from input parameters simulating specific phenes, such as gravitropic setpoint angle or lateral root initiation interacting with environmental conditions. For example, as a result of spatio‐temporal heterogeneity in soil nutrient availability, growth angles may differentially affect P and N uptake, but also affect the degree of inter‐ vs intra‐plant root competition (Ge *et al*., 2000; Rubio *et al*., 2001; Dathe *et al*., 2013). The results of simulated maize–bean–squash intercropping systems showed that RSA and N fixation (bean) work towards reduced competition and increased biomass (Postma & Lynch, 2012; Zhang *et al*., 2014). Competition among branches of the same parent root may become stronger when the root branching density increases, and, as this increase results in greater sink strength, but not greater source strength (in C available for growth), the individual roots may remain shorter. By simulating these processes, Postma *et al*. (2014a) estimated that the optimal branching density (assuming that parent roots have the same root branching density) for maize was lower when N availability decreased. The benefit of fewer but longer laterals in low‐N soils was confirmed in a genotypic contrast study (Zhan *et al*., 2015). Walk *et al*. (2006) estimated the tradeoffs between basal root growth and adventitious root growth in bean, and concluded that adventitious roots might be of most benefit when P availability is low. Although these RSA traits represent tradeoffs, other traits may work in synergy towards greater productivity on low‐nutrient soils (Ma *et al*., 2001; Postma & Lynch, 2011a; Miguel *et al*., 2015). OpenSimRoot has also increased our understanding of how integrated phenotypes function. This was demonstrated by York *et al*. (2015), who used simroot to estimate how changes in maize RSA, introduced by breeding over 100 yr, might affect the nutrient uptake efficiency of modern cultivars. New functionality described here will enable new studies of the function of whole plant traits, such as tiller formation and its influence on RSA.

### Relationships between RSA traits and root system descriptors

Many researchers have determined what might be called geometric descriptors of RSA: root length density profiles, fractal dimension, specific root length, total root length, rooting depth and convex‐hull (Fitter & Stickland, 1992; Clark *et al*., 2011). These descriptors can be computed on simulated roots, and their relation to architectural, anatomical or functional traits can be inferred. For example, differences in the specific root length of a root system may be related to anatomical changes, or a different ratio of thick to finer roots. Nielsen *et al*. (1997) determined differences in fractal dimensions between P‐efficient and P‐inefficient genotypes, and Walk *et al*. (2004) applied simroot to show how soil exploration for P was related to the fractal dimensions of the root system. Miguel *et al*. (2015) applied simroot to perform ‘virtual coring’ in order to support the idea that genotypic differences in rooting depth might best be seen when coring in between rows. These studies show how the geometric aspects of the root system can be related to root traits and function, something not easily derived from empirical measurements of actual root systems.

### Scaling up from root anatomy to crop

At its smallest spatial scale, OpenSimRoot represents root anatomy, and, at its largest scale, it simulates crop measures, such as biomass, nutrient uptake and root zone depletion and leaching. For example, Ma *et al*. (2001) focused on root hairs in *Arabidopsis thaliana* and concluded that their length and density contribute synergistically towards greater P uptake. Chen *et al*. (2011, 2013) used simroot and lupin phenotypic data to compute that the contribution of root hairs to total P uptake might vary strongly among genotypes. Postma & Lynch (2011a,b) and Schneider *et al*. (H. M. Schneider, J. A. Postma, T. Wojciechowski, C. Kuppe, J. P. Lynch, unpublished) simulated the root class‐ and time‐dependent formation of RCA and root cortical senescence (RCS), respectively, and determined that RCA and RCS may be mechanisms underlying greater growth on low‐nutrient soils in maize, bean and barley, possibly via efficient use and recycling of resources. Genotypic contrast studies on low‐N soils concur with these simulation results (Saengwilai *et al*., 2014), which suggests that OpenSimRoot can be used to scale up from anatomy to crop stands.

### OpenSimRoot as an extension to plant phenotyping

Technologies such as X‐ray CT and MRI have been adapted to image root systems non‐destructively and provide non‐invasive ways to phenotype whole root systems in 3D in soil (Mooney *et al*., 2012; Mairhofer *et al*., 2013; van Dusschoten *et al*., 2016). The utility of feeding such data to a model was demonstrated by Stingaciu *et al*. (2013) for a non‐growing lupin root system. Using time estimates, OpenSimRoot can simulate the growth of a root system, such that the RSA is identical to that imaged. Figure 6(a,b) (for animation, see Movie S1) shows an MRI image and the simulated root system. The simulation does not include a small portion (*c*. 8%) of the roots visible in the 3D image data because of limitations in image segmentation, rather than in the model. OpenSimRoot can add ‘MRI‐non‐visible’ finer roots to the simulation according to existing model rules, and the simulation can be extended beyond the measured time, to predict continued growth of the root system. Importantly, OpenSimRoot modules for nutrient and water uptake can be enabled with the architectural phenotypes derived from measurements and simulation, and functions can be ascribed to the traits. This may help researchers and breeders go from the image to a functional understanding of the measured root systems, and to compare genotypes, not only on the basis of geometry, but also on the basis of modeled ability to take up water and nutrients. For example, Fig. 6(c,d) shows a CT image and corresponding OpenSimRoot simulation of nitrate depletion zones around the root system. Integration of the model into phenotyping pipelines is also likely to help reveal deficits of the model, and to provide modelers with a basis for improving parameterization and/or algorithms. This important development considerably widens the scope of application of OpenSimRoot.

## Discussion

We have described the first open‐source version of the RSA model simroot, which is now available for use by biologists and modelers. New features that expand its use include hydrology to simulate and understand root system hydraulic properties. A novel area of application includes the simulation of non‐invasive 3D phenotypic data of RSA from MRI and X‐ray CT, and their putative functions in nutrient and water uptake. To our knowledge, OpenSimRoot is currently the most feature‐rich and widely published multiplatform RSA model (Dunbabin *et al*., 2013), which is freely available for direct download (http://rootmodels.gitlab.io/OpenSimRoot). The new open‐source implementation combines features that will enable the expansion of use for plant and crop science:

- a modular, plugin infrastructure for extension of the model;
- a default predictor‐corrected numerical scheme for integration and coupling;
- the ability to predefine any data that are measured, in which the model will use the measured data instead of its algorithm for simulation (e.g. the root system, and optionally its history, may be partly predefined based on MRI or CT images);
- integration with a shoot model;
- ability to simulate competition among plants of different species;
- maintained by an international community of root researchers.

Relationships in crop models that are typically only defined empirically, such as competition among roots for nutrients, or root length density profiles, are actually a result of RSA, and therefore RSA models provide insight into relations between measurable traits and emerging properties at the crop level. We regard the heuristic value of the model, and its use as a tool for the development and testing of concepts, and the prediction of mechanisms and trends, as the more important motivation for model studies with, and continued development of, OpenSimRoot. The model may have further utility in extending phenotyping pipelines by estimating genotype performance based on measured root phenotypes.

Future development will be community driven, and may include new processes, such as root signaling networks, drought responses, soil microbial interactions and soil chemistry. As our mechanistic understanding of different processes increases, OpenSimRoot's hierarchical structure will allow new empirical data to be represented by new algorithms. For example, gravitropism may be simulated on the basis of the understanding of differential cell elongation, rather than on the current empirically derived input. Open sourcing allows other modelers to couple OpenSimRoot to their models. For example, shoot architectural models might be coupled to OpenSimRoot in order to understand competition for light and shoot architectural traits in relation to RSA traits. Finally, opening up the code enables developers of other RSA models to compare the results of OpenSimRoot with those of their models, which may lead to constructive critique and improvements of all RSA models and, by extension, discoveries for improvements in our understanding of plant and crop resource efficiencies.

## Author contributions

J.A.P. and M.W. planned the manuscript. J.P.L. conceived of simroot and led its development through 2011. J.A.P. rewrote the code, expanded its capabilities and has led its development since 2011, with mathematical support from C.K. since 2013. J.A.P., C.K., N.M. and M.R.O. programmed various parts of the model code. All authors were involved in open sourcing of the code and forming a development team. M.J.B., N.M., M.G. and M.R.O. contributed the CT image data and the simulation output based on that data. J.A.P., C.K., M.W., J.P.L., M.R.O. and M.J.B. wrote various parts of the manuscript, with input from all authors.

## Supporting information

Please note: Wiley Blackwell are not responsible for the content or functionality of any Supporting Information supplied by the authors. Any queries (other than missing material) should be directed to the *New Phytologist* Central Office.

**Notes S1** Description of the simulabase application programming interface (API).
**Notes S2** How to run OpenSimRoot: description of the command line interface (CLI).
**Notes S3** Overview of all classes that form OpenSimRoot, including list of plugins.
**Notes S4** Example C++ code for a plugin.
**Notes S5** Technical description of water and nutrient modules.
**Notes S6** Example input file.
**Notes S7** Example graph of state variables and their dependences.

**Movie S1** Animation of Fig. 6.
