# Supplementary material for: OpenSimRoot: widening the scope and application of root architectural models
Source: New Phytol. 2017 Jun 27;215(3):1274–86. doi: 10.1111/nph.14641 (PMC5575537; doi:10.1111/nph.14641)
Supplement: Supplementary file 1 — Notes S1 Description of the simulabase application programming interface (API). Notes S2 How to run OpenSimRoot: description of the command line interface (CLI). Notes S3 Overview of all classes that form OpenSimRoot, including list of plugins. Notes S4 Example C++ code for a plugin. Notes S5 Technical description of water and nutrient modules. Notes S6 Example input file. Notes S7 Example graph of state variables and their dependences. [file NPH-215-1274-s001.pdf]

New Phytologist Supporting Information

Article title: *OpenSimRoot*: Widening the scope and application of root architectural models

Authors: Postma, Johannes A.[1], Kuppe, Christian[1] , Owen, Markus R.[2,3], Mellor, Nathan[3,4], Griffiths, Marcus[3,4], Bennett, Malcolm J.[3,4], Lynch Jonathan P.[3,4,5], Watt, Michelle[1]

1) Plant Sciences, Institute of Bio and Geosciences 2, Forschungszentrum Jülich, Wilhelm-Johnen Straße 52425 Jülich, Germany
2) Centre for Mathematical Medicine and Biology, School of Mathematical Sciences, University of Nottingham, UK
3) Centre for Plant Integrative Biology, University of Nottingham, UK
4) Plant & Crop Sciences Division, School of Biosciences, University of Nottingham, UK
5) Department of Plant Science, Pennsylvania State University, USA

Article acceptance date: 04/26/17

The following Supporting Information is available for this article:

Note S1 Description of the SimulaBase API

Note S2 How to run *OpenSimRoot*: description of CLI

Note S3 Example C++ code for a plugin

Note S4 Example C++ code for a plugin

Note S5 Technical description of water and nutrient modules

Note S6 Example input file

Note S7 Example graph of state variables and their dependencies

Movie S1 Animation of Figure 6b.

Note S1: Application programming interface (API) of the SimulaBase class

This interface is used by the plugins to navigate the hierarchy and retrieve necessary data. For an example see, Note S4. Developers that would like to develop a new plugin, will need this interface in order to retrieve data from other minimodels. These minimodels are in a hierarchy. The methods listed here can be used to find those minimodels in the hierarchy, and to request data from them. Minimodels are instantiations (objects) of class (type) SimulaBase.

//Method to retrieve meta data on a given minimodel such as its name, path in the hierarchy, lifetime of the object, and its units.

```
std::string getName()const; //name of object
std::string getPrettyName()const; //some what more humen readable name
std::string getPath()const; //path to the object
virtual std::string getType()const; //What type this object has
bool evaluateTime(const Time &t)const; //check if t is within lifetime
Time getEndTime()const; //get the end time of object
Time getStartTime()const; //get the start time of object
virtual Unit getUnit(); //get the unit
void checkUnit(const Unit& unit)const; //check if unit equals given unit
void setUnit(const Unit &newUnit); //change unit
virtual void getXMLtag(Tag &tag); //get the object as tag (xml output)
```

//Methods to navigate the minimodel hierarchy

The difference between the get() and existing() methods is that when the object does not exist get() will throw an error and terminate the simulation, whereas existing() will return a NULL pointer. The getPath() methods will navigate a symbolic path just as a path in a filesystem is navigated. For example
getPath("../mySib") translates to getSibling("mysib"), where the later is more efficient.

```
SimulaBase* getParent()const;
SimulaBase* getParent(const unsigned int i) const;
int getNumberOfChildren()const;//does not update!
int getNumberOfChildren(const Time &t);//does update
SimulaBase* getChild(const std::string & name,const Time & t);
SimulaBase* existingChild(const std::string & name,const Time & t);
SimulaBase* getChild(const std::string & name);
SimulaBase* existingChild(const std::string & name);
SimulaBase* getChild(const std::string & name,const Unit & u);
SimulaBase* existingChild(const std::string & name,const Unit & u);
SimulaBase* getSibling(const std::string & name,const Time & t);
SimulaBase* existingSibling(const std::string & name,const Time & t);
SimulaBase* getSibling(const std::string & name);
SimulaBase* existingSibling(const std::string & name);
SimulaBase* getSibling(const std::string & name,const Unit & u);
SimulaBase* existingSibling(const std::string & name,const Unit & u);
//Sibling can be retrieved in alphabetic order.
SimulaBase* getNextSibling(const Time &t);
SimulaBase* getNextSibling()const;
SimulaBase* getPreviousSibling(const Time &t);
```

```
SimulaBase* getPreviousSibling()const;
SimulaBase* getFirstChild(const Time &t);
SimulaBase* getFirstChild()const;
SimulaBase* getLastChild()const;

SimulaBase* getPath(const std::string &name);
SimulaBase* getPath(const std::string &name, const Time &t);
SimulaBase* getPath(const std::string &name, const Unit &u);
SimulaBase* existingPath(const std::string &name);
SimulaBase* existingPath(const std::string &name, const Time &t);
SimulaBase* existingPath(const std::string &name, const Unit &u);

typedef std::vector<SimulaBase*> List;
void getAllChildren(List&, const Time &t);
void getAllChildren(List&)const;
```

//Method for walking along a root axis. Retrieves the minimodel with the same

```
name associated with the next vertex.
virtual SimulaBase* followChain(const Time & t);
```

//Methods to retrieve specific subsets of minimodels based on position

```
typedef  std::multimap<Coordinate,SimulaBase*> Positions;
static void getAllPositions(const Time & t, Positions& list);
static void getAllPositions(Positions& list);
void getYSlice(const Time &, const double, const double, Positions&);
void getPositionsWithinRadius(const Time &, const Coordinate& c, const
double & r, Positions&);
void getPositionsInsideBox(const Time &, const Coordinate&, const
Coordinate &, Positions&);
```

//Methods for retrieving data

```
virtual void get(const Time &t, int &returnConstant);
virtual void get(const Time &t, std::string &returnConstant);
virtual void getRate(const Time &t, Time &var);
virtual void get(const Time &t, Coordinate &point);
virtual void get(const Time &t, MovingCoordinate &point);
virtual void getAbsolute(const Time &t, Coordinate &point);
virtual void getBase(const Time &t, Coordinate &point);
virtual void getRate(const Time &t, Coordinate &point);
virtual void getAbsolute(const Time &t, MovingCoordinate &point);
virtual void get(int &returnConstant);
virtual void get(std::string &returnConstant);
virtual void get(bool &returnConstant);
virtual void get(const Time &x, Time &y);
virtual void get(Time &x);
virtual void get(const Time &t, const Coordinate & pos, double &y);
virtual void get(const Time &t, const Coordinate & pos, Coordinate &y);
virtual void getRate(const Time &t, const Coordinate & pos, double &y);
virtual void get(Coordinate &point);
virtual void getAverageRate(const Time &t1, const Time &t2, double &var);
```

```
virtual void getAverageRate(const Time &t1, const Time &t2, Coordinate
&var);

//reverse data look up: returns time that object was nearest to given
value or position. Only works if the object is not garbage collected
virtual void getTime(const Coordinate &p, Time &t, Time tmin=-1, Time
tmax=0);
virtual void getTime(const double &p, Time &t, Time tmin=-1, Time tmax=0);
```

//Method for setting data, probably only implemented for timetables.

```
virtual void set(const double &x, const double &y);
```

//Methods to retrieve info on timestepping of a minimodel

```
virtual Time &minTimeStep();
virtual Time &maxTimeStep();
virtual Time &preferedTimeStep();
virtual Time lastTimeStep();
```

//Methods to control garbage collection, which will basically clean up the simulation history

```
virtual void collectGarbage(const Time&); //clean up history
virtual void garbageCollectionOff(); //keep history of this object always
```

//Other methods

```
void stopUpdatefunction(); //When implementing an objectgenerator signal
it has
finished creating all objects for all times, and can be deleted.
static void updateAll(const Time &); //update whole tree
void updateRecursively(const Time &); //update subtree
static void signalMeAboutNewObjects(SimulaBase* me); //if plugin has the
addObject() implemented, it will be signaled when new objects are being
instantiated by any of the object generators.
```

Note S2: Command line interface (CLI) of OpenSimRoot: How to run and use the model

OpenSimRoot has a command line interface, which means that you operate the model from a terminal using commands, not with a graphical interface and the mouse.

Step 1: Open a terminal (under windows 10 you may use the program named CMD)

Step 2: Go to the folder where you want to run the model, use the command cd to navigate, for example: cd MyRunFolder

Step 3: We assume that the folder contains the OpenSimRoot executable. With the “ls” command you can list all folders (or on windows the command is “dir”). Here we see that my folder contains the executable OpenSimRoot (conveniently made green, as it is executable) and a XML input file.

```
$ ls
OpenSimRoot  SimpleCropModel.xml
$
```

Step 4: OpenSimRoot has a small build in help which we we can run by typing ./OpenSimRoot -h (on windows you do not type the path “./” in front of the executable).

```
$ ./OpenSimRoot -h
Usage: OpenSimRoot [OPTIONS] [FILE]
OpenSimRoot simulates a model defined in FILE

  -f, --file                   specify simulation file, default last argument
  -h, --help or /?             print this help message
  -v                           be verbose with warnings
  -q                           be quite with warnings
  -l, --list                   print list of registered functions
  -V, --verify                 Experimental function for verifying input files

Examples:
  OpenSimRoot -h               Prints this message
  OpenSimRoot runModel.xml     Runs the SimRoot model

Support at j.postma@fz-juelich.de
Licensed to you under the GPLv3 license.

There are no warnings.
Simulation took (hours:minutes:seconds): 0:0:0
$
```

The help shows how to run OpenSimRoot, and gives you some options and their explanation.

Step 5: Like the help shows, running the model is done by appending the input file: ./OpenSimRoot SimpleCropModel.xml

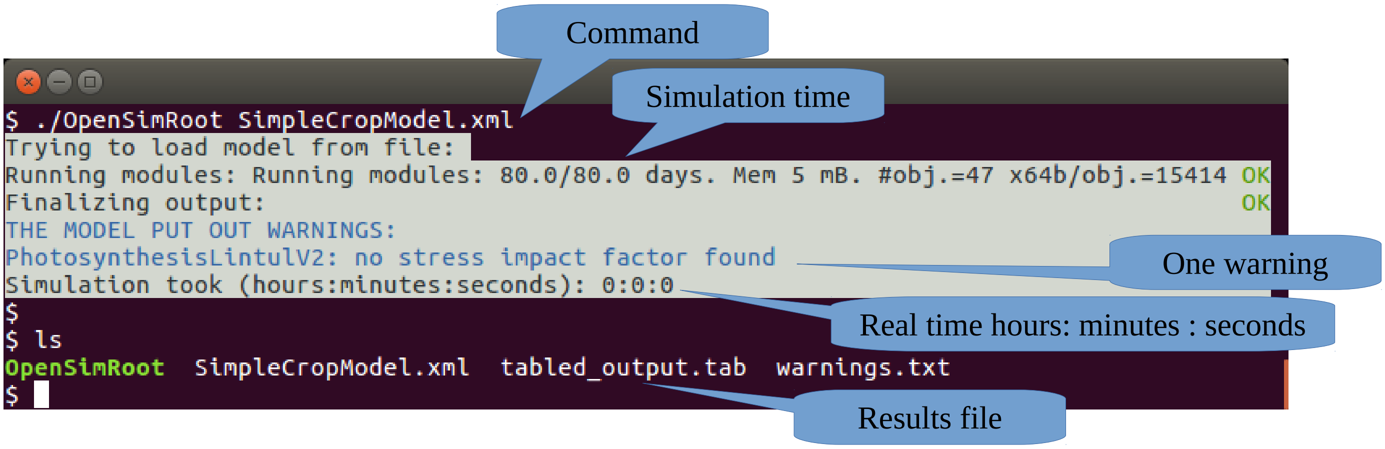

Again with ls (dir) you can list the filer, the model created two new files, one containing warnings, one containing the simulation results.

Step 6: The results of the simulation are in the tabled_output.tab file which can be viewed with any program that opens text files. Here we simply show the first lines with the command head:

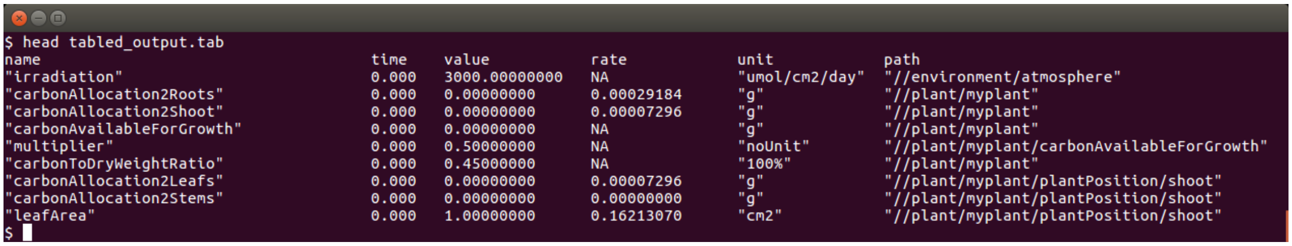

The file contains a header in the first row, and 6 columns listing the name of the state variable, the time, the value, the rate of change of that state variable (if simulated), the unit of the state variable, and the path in the hierarchy to this state variable.

Step 7: The tabled_output.tab file is also easily imported into a spreadsheet program. By enabling the auto filter and selecting leafArea, we can easily create a plot.

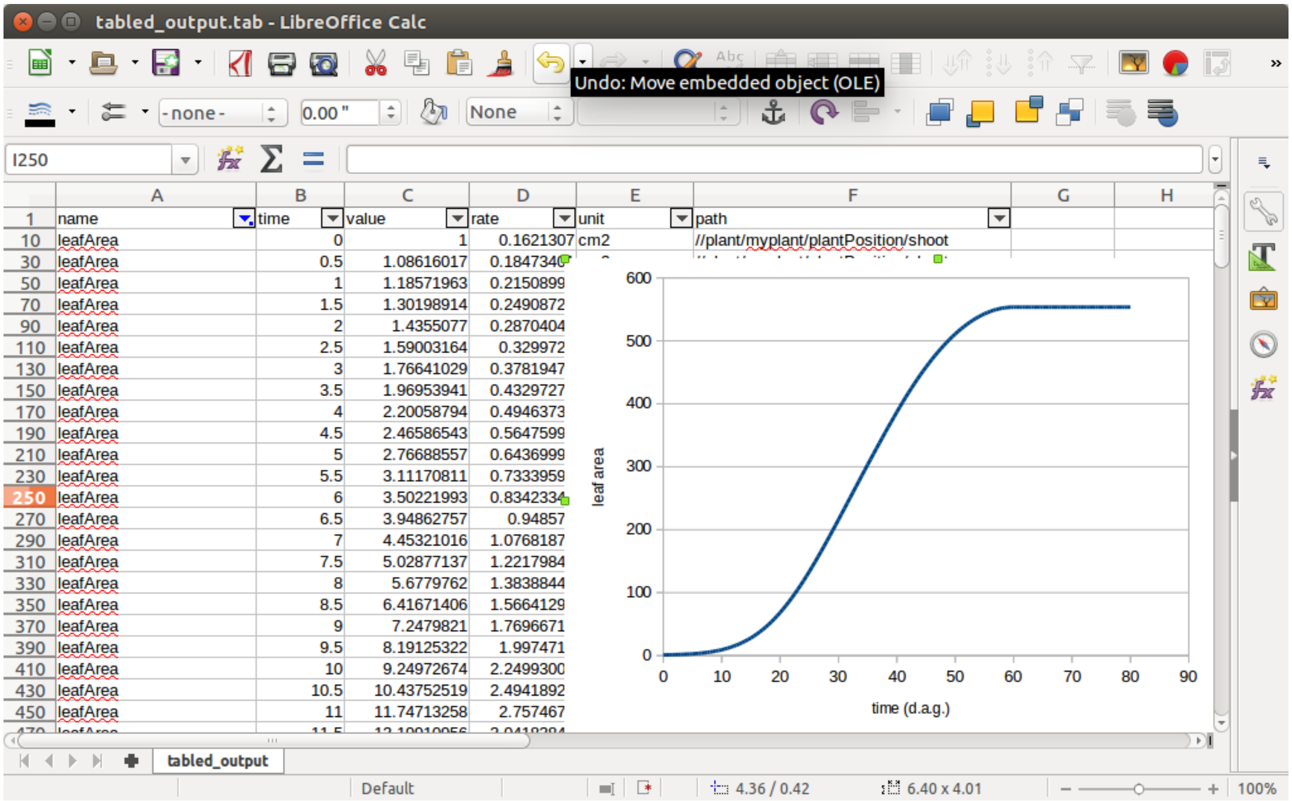

8. The same can be achieved in R using this script:

```
d<-read.table("tabled_output.tab",header=T)
f=d$name=="leafArea"
plot(value~time,data=d[f,],ylab=~"leaf area (cm"^2*")", xlab="time (d.a.g)")
```

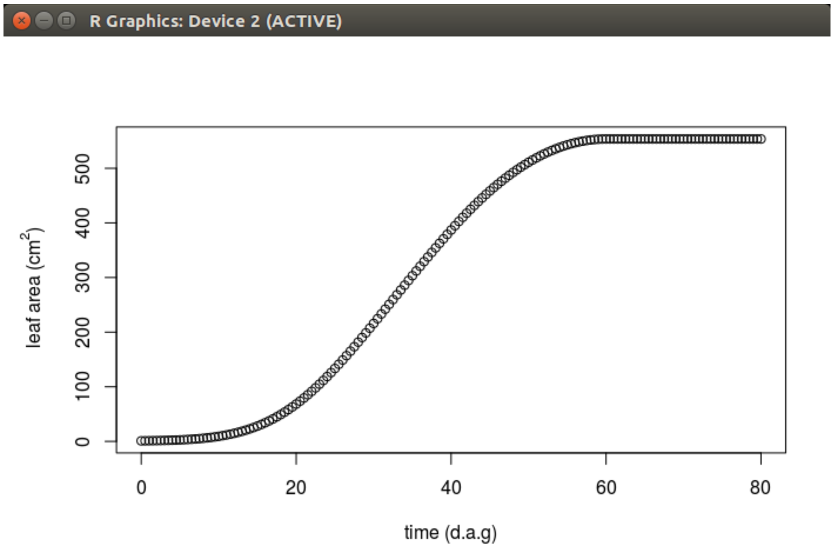

step 9: Editing the input file can be done with any text file editor. Here I opened the file with the command nano tabled_output.tab and the result is an xml formatted file in which we can change the numbers, save and rerun. In white you see the numbers, and scrolling to the bottom you would see more.

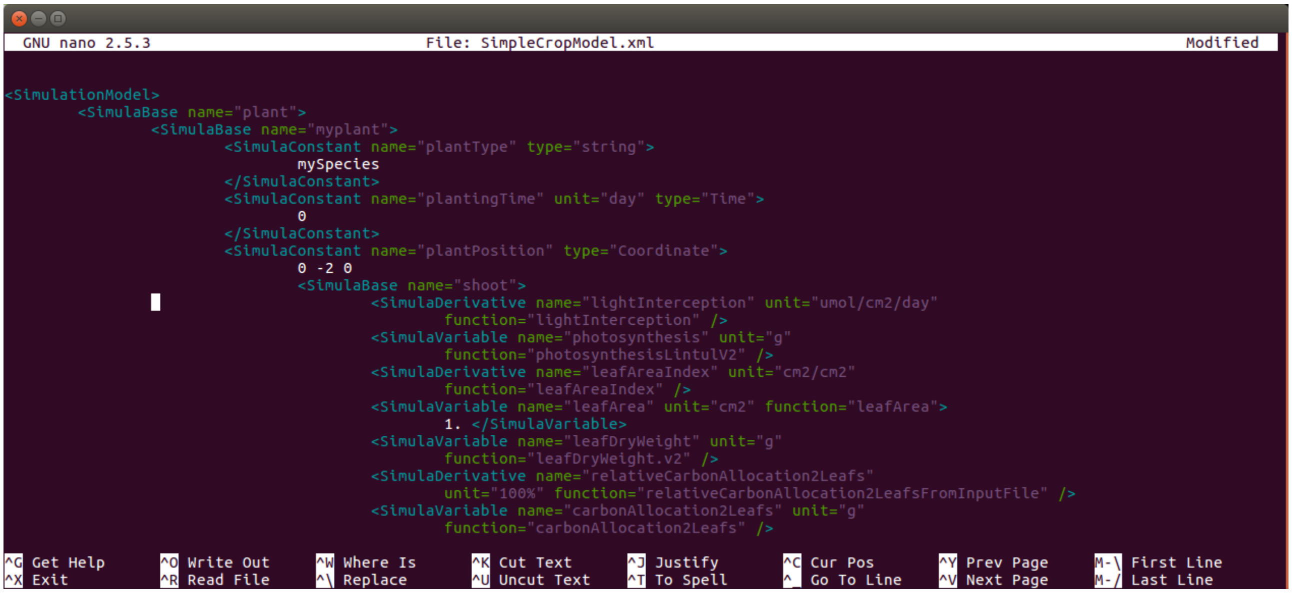

step 10: You see several functions listed that are used to simulate a state variable. To get a list of all functions that are included in your OpenSimRoot version use the command OpenSimRoot -l. This will list all plugins that are included with OpenSimRoot.

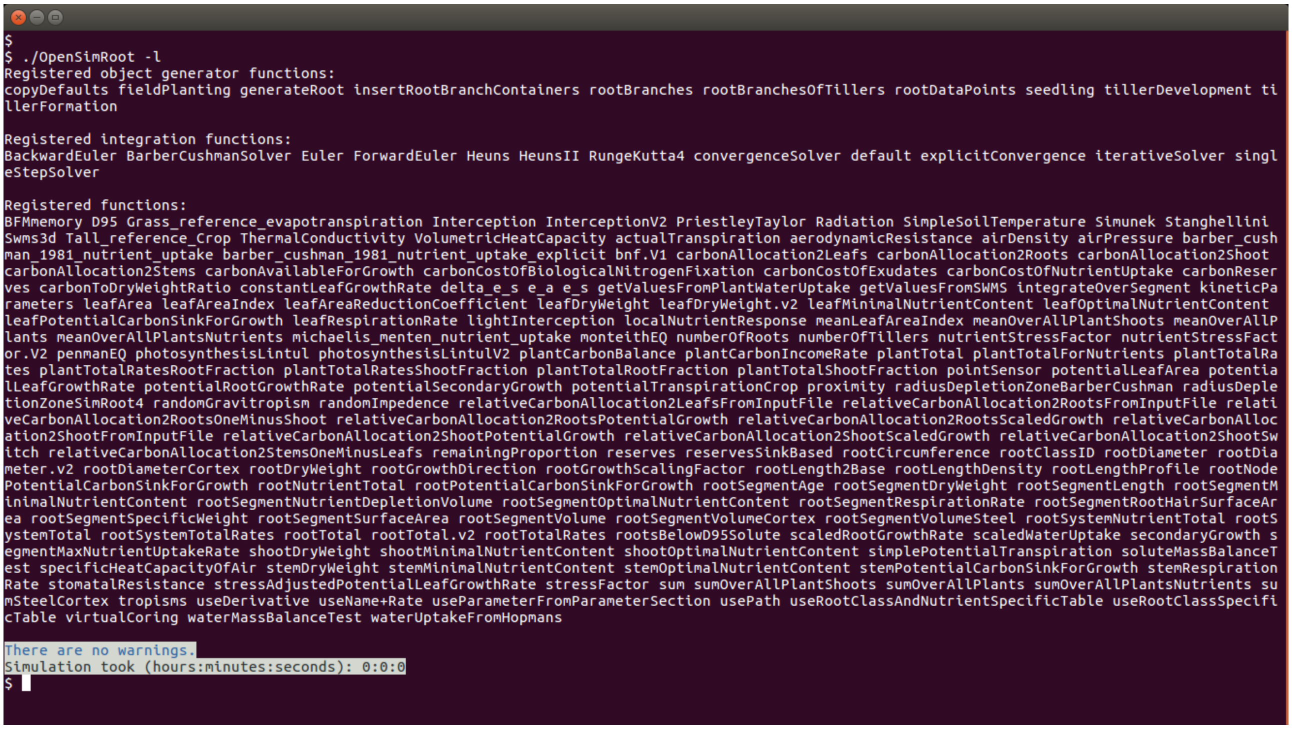

Note S3: Class hierarchy of OpenSimRoot code

This document lists the class hierarchy for the most important classes in OpenSimRoot.

Minimodels

Minimodels are of type SimulaBase and encapsulate one time and location dependent state variable. The inhertance diagram for all SimulaX classes is given in Figure S3.1.

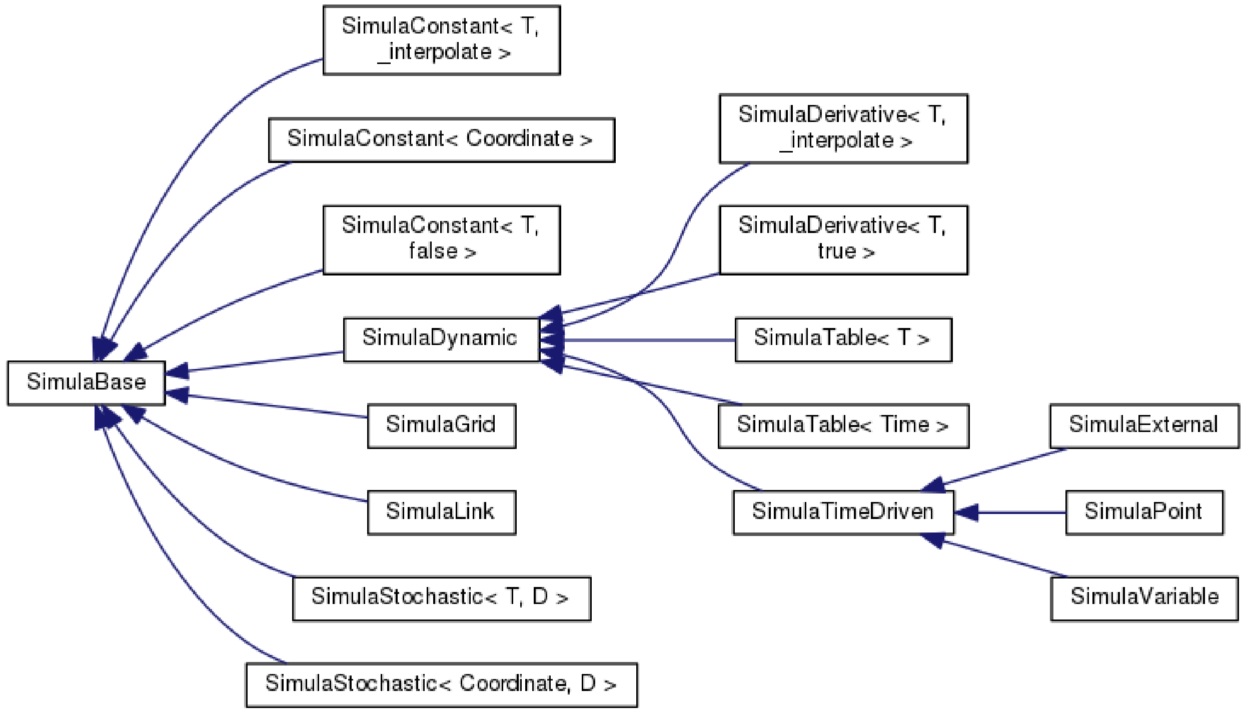

Figure S3.1: Inheritance diagram for all SimulaBase classes.

- SimulaConstant encapsulates a constant of various types.
- SimulaDerivative encapsulates an algorithm. Available algorithms are all the DerivativeBase derived plugins.
- SimulaTable encapsulate an array of time,value combinations. Values are interpolated.
- SimulaExternal provides a mechanism for encapsulating other dynamic simulation models.
- SimulaPoint simulates a point and its movement through space.
- SimulaVariable simulates a value and change over time using numerical integration.
- SimulaGrid simulates a static, 3D field using a list of Coordinates with values and a 3D interpolation algorithm
- SimulaLink simply bridges to another minimodel in the hierarchy of minimodels.
- SimulaStochastic draws numbers from a random number generator.

Inherited from DerivativeBase

Below is a list of all the plugins that directly, or indirectly, inherit from DerivativeBase and can be used by SimulaVariable, SimulaPoint or SimulaDerivative for computation.

ActualTranspiration
ActualVaporPressure
AerodynamicResistance
AirDensity
AirPressure
BFMmemory
BiologicalNitrogenFixation
CarbonAllocation2Leafs
CarbonAllocation2Roots
CarbonAllocation2Shoot
CarbonAllocation2Stems
CarbonAvailableForGrowth
CarbonCostOfBiologicalNitrogenFixation
CarbonCostOfNutrientUptake
CarbonReserves
CinDryWeight
ConstantRootGrowthRate
D95
ETbaseclass
Grass_reference_evapotranspiration
Penman
PenmanMonteith
PriestleyTaylor
Stanghellini
Tall_reference_Crop
GetValuesFromPlantWaterUptake
GetValuesFromSWMS
Imax
Interception
InterceptionV2
LeafArea
LeafAreaIndex
LeafAreaReductionCoefficient
LeafDryWeight
LeafDryWeight2
LeafPotentialCarbonSinkForGrowth
LeafRespirationRate
LightInterception
LocalNutrientResponse
MeanLeafAreaIndex
NumberOfRoots
NumberOfTillers
NutrientStressFactor
NutrientStressFactorV2
PhotosynthesisLintul
PhotosynthesisLintulV2
PlantCarbonBalance
PlantCarbonIncomeRate
PlantTotal
PointSensor
PotentialLeafArea
PotentialTranspirationCrop
Proximity
Radiation
RadiusDepletionZoneBarberCushman
RadiusDepletionZoneSimRoot4
RandomGravitropism
RandomImpedence
RelativeCarbonAllocation2LeafsFromInputFile
RelativeCarbonAllocation2RootsFromInputFile
RelativeCarbonAllocation2RootsOneMinusShoot
RelativeCarbonAllocation2RootsPotentialGrowth
RelativeCarbonAllocation2RootsScaledGrowth
RelativeCarbonAllocation2ShootFromInputFile
RelativeCarbonAllocation2ShootPotentialGrowth
RelativeCarbonAllocation2ShootScaledGrowth
RelativeCarbonAllocation2ShootSwitch
RelativeCarbonAllocation2StemsOneMinusLeafs
RemainingProportion
Reserves
ReservesSinkBased
RootCircumference
RootClassID
RootDryWeight
RootGrowthDirection
RootGrowthScalingFactor
RootLength2Base
RootLengthDensity
RootLengthProfile
RootNodePotentialCarbonSinkForGrowth
RootPotentialCarbonSinkForGrowth
RootsBelowD95Solute
RootSegmentAge
RootSegmentRespirationRate
RootSegmentRootHairSurfaceArea
RootSegmentSpecificWeight
RootSystemTotal
RootTotal
RootTotal2
SaturatedVaporPressure
ScaledRootGrowthRate
ScaledWaterUptake
ShootDryWeight
ShootOptimalNutrientContent
SimplePotentialTranspiration
SimpleSoilTemperature
SlopeVaporPressure
SoluteMassBalanceTest
SpecificHeatCapacityOfAir
StemDryWeight
StemPotentialCarbonSinkForGrowth
StemRespirationRate
StomatalResistance
StressAdjustedPotentialLeafArea
StressFactor
SumCarbonCosts
SumOverPlants
SumOverPlantsShoot
SuperCoring
Swms3d
ThermalConductivity
TotalBase
CarbonCostOfExudates
CortexDiameter
IntegrateOverSegment
PotentialSecondaryGrowth
RootDiameter
RootSegmentDryWeight
RootSegmentLength
RootSegmentSurfaceArea
RootSegmentVolume
RootSegmentVolumeCortex
SecondaryGrowth
SumSteelCortex
TotalBaseLabeled
Barber_cushman_1981_nutrient_uptake
Barber_cushman_1981_nutrient_uptake_explicit
MichaelisMenten
OptimalNutrientContent
RootSegmentNutrientDepletionVolume
SegmentMaxNutrientUptakeRate
Tropisms
UseDerivative
UseParameterFromParameterSection
UseRootClassAndNutrientSpecificTable
VolumetricHeatCapacity
WaterMassBalanceTest
WaterUptakeFromHopmans

List of plugins for simulating various processes

Note that these are a list of classes, as they appear in the code. Registration of the plugins may occur under different names. Inputfiles use the registered names, not the class names. Use OpenSimRoot -l to get that list. See also operation manual in Note S2.

Integration functions

The SimulaVariable and SimulaPoint classes use helper functions for integrating the result. Several integration methods have been implemented (Figure S3.2). New integration functions can be added and registered, using the plugin framework, similar to the classes that inherit from DerivativeBase.

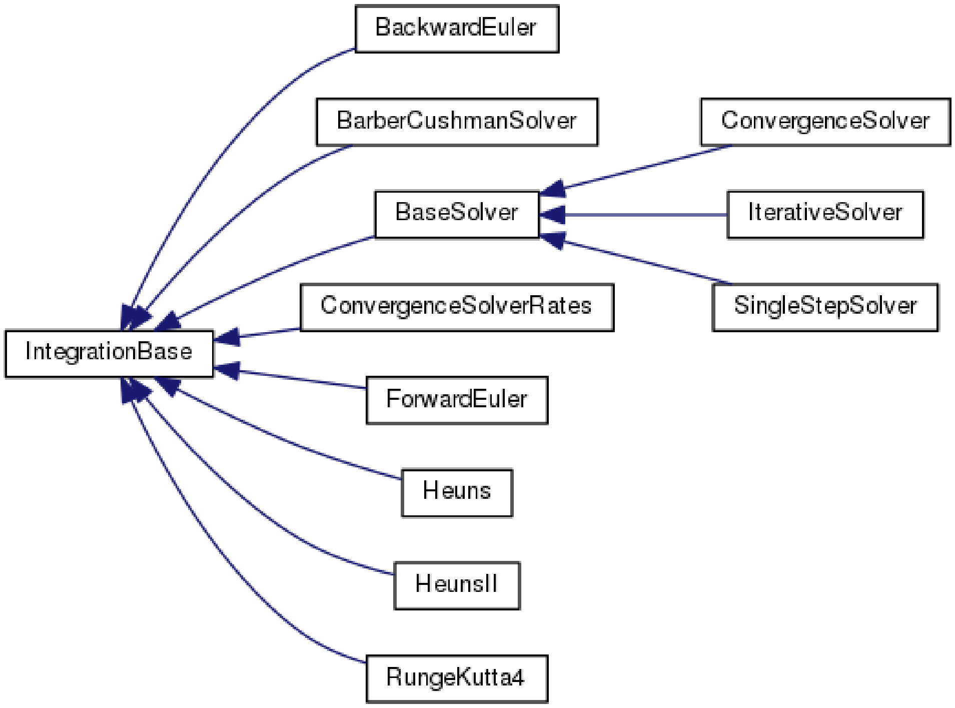

Figure S3.2: Inheritance diagram for the integration classes

Object generators

Object generators are plugins that can be associated with any SimulaX object and update the list of children when a child is requested.

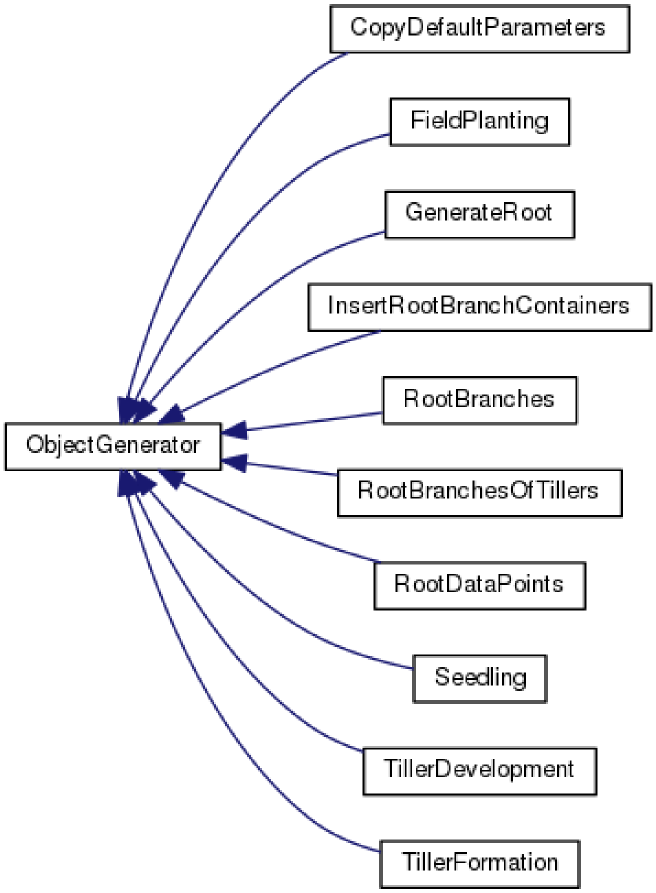

Figure S3.3: Inheritance diagram for the object generators

Note S4: Plugin example code

Here we give example code for a simple plugin and the code needed to register this plugin with OpenSimRoot. Once the code has been put into a text file, it can be compiled and linked to OpenSimRoot.

1) For new algorithms

```
//Class declaration. Class should inherit from DerivativeBase, have a constructor, and
implements two virtual methods, getName() and calculate(). The example class presented here has
two SimulaBase pointers as private members, which will be used to connect to the minimodels that
simulate length and diameter of a root segment and to retrieve their values.

class RootSegmentSurfaceArea:public DerivativeBase{
public:
        RootSegmentSurfaceArea(SimulaDynamic* pSD);
        std::string getName()const;
protected:
        void calculate(const Time &t,double &var);
private:
        SimulaBase *diameter,*length;
};

//the constructor of our class. pSD is the pointer to the minimodel that uses the plugin for
computation
RootSegmentSurfaceArea::RootSegmentSurfaceArea(SimulaDynamic* pSD):DerivativeBase(pSD)
{
//We check that the user set the unit right
        pSD->checkUnit("cm2");
//We retrieve the pointers
        length=pSD->getSibling("rootSegmentLength","cm");
        diameter=pSD->getSibling("rootDiameter","cm");
}

//the computation
void RootSegmentSurfaceArea::calculate(const Time &t,double &area){
//first we retrieve data
        double d,l;
        diameter->get(t,d);
        length->get(t,l);
//second we compute
        area=l*d*PI;
}

//the  name under which the plugin will be registered, make sure it is unique, use OpenSimRoot
-l to see what names are already taken
std::string RootSegmentSurfaceArea::getName()const{
        return "rootSegmentSurfaceArea.v3";
}

//Now we create a function for instantiating our class
DerivativeBase * newInstantiationRootSegmentSurfaceArea(SimulaDynamic* const pSD){
   return new RootSegmentSurfaceArea(pSD);
}

//And we register this plugin using a static instantiation of a class which guarantees that the
constructor is when OpenSimRoot is started
static  class AutoRegisterMyNewPlugin {
public:
   AutoRegisterMyNewPlugin() {
//this line does the registration. Make sure you register under the same name as the getName()
method returns. This important for the model dump being loadable again.
        BaseClassesMap::getDerivativeBaseClasses()["rootSegmentSurfaceArea.v3"] =
newInstantiationRootSegmentSurfaceArea;
} rf9843hh923h; //the one static instance of this class
```

2) For new integration functions

```
//class declaration, must inherit from IntegrationBase, has a constructor,
// a getName() method and at least one integrate method
class BackwardEuler:public IntegrationBase{
public:
  BackwardEuler();
  std::string getName()const;
protected:
  virtual void integrate(SimulaVariable::Table & data, DerivativeBase & rateCalculator);
  virtual void integrate(SimulaPoint::Table & data, DerivativeBase & movementCalculator);
};

BackwardEuler::BackwardEuler():IntegrationBase(){}

void BackwardEuler::integrate(SimulaVariable::Table & data, DerivativeBase &rateCalculator){
  //...Your new algorithm here which should extend the data table, the derivative (rates) that
should be used are retrieved from the rateCalculator. For examples see code.
}

void BackwardEuler::integrate(SimulaPoint::Table & data, DerivativeBase & movementCalculator){
  //...Your new algorithm here, but then suitable for Coordinates, not doubles. Intended to
allow the simulation of a point moving through space. Mostly used to simulate the growth
trajectory of the root tip
};

std::string BackwardEuler::getName()const{
  return "BackwardEuler";
}

//function for instantiating the class
IntegrationBase * newInstantiationBackwardEuler(){
    return new BackwardEuler();
}

//Register the instantiation function
static class AutoRegisterIntegrationFunctions {
public:
  AutoRegisterIntegrationFunctions() {
    BaseClassesMap::getIntegrationClasses()["BackwardEuler"] = newInstantiationBackwardEuler;
  }
}p44608510843540385;//the one static instance of this class
```

3) For object generators

```
//class declaration for an object generator
class MyGenerator: public ObjectGenerator {
public:
  void initialize(const Time &t);
  void generate(const Time &t);
  MyGenerator(SimulaBase* const pSB);
};

//construction is delayed. Code is in the initialize method
MyGenerator::MyGenerator(SimulaBase* const pSB) :
  ObjectGenerator(pSB) {
}

//collecting of info, and or construction of minimodels at the start of the simulation
void MyGenerator::initialize(const Time &t) {
  //collect some info about planting time
  Time plantingTime;
  SimulaBase *pt=pSB->existingChild("plantingTime");
  if (pt) {
    //read planting time from file
    pt->get(t, plantingTime);
  } else {
    //copy from parent
    plantingTime = pSB->getStartTime();
  }

  //generate new plant by copying the template
  pSB->copyAttributes(plantingTime, ORIGIN->getChild("plantTemplate"));

  //we are done
  pSB->stopUpdatefunction();
}

void MyGenerator::generate(const Time &t) {
  //add code if there is time dependent generation of objects, not just at the start
}

//the function for instantiation of the class
ObjectGenerator * newInstantiationMyGenerator(SimulaBase* const pSB) {
  return new MyGenerator(pSB);
}

//register the instantiation function
static class AutoRegisterMyGeneratorInstantiationFunctions {
public:
  AutoRegisterMyGeneratorInstantiationFunctions() {
    BaseClassesMap::getObjectGeneratorClasses()["MyGenerator"] =
      newInstantiationMyGenerator;
  }
} p4595582386;
```

Note S5: Detailed description of the water and nutrient submodules

Watermodule

Plant transpiration is simulated by OpenSimRoot, assuming that water availability is not limiting and stomatal conductance is constant. Transpiration and evaporation need to be separated within OpenSimRoot. Transpiration can be estimated from a fixed water use efficiency parameter (which simply links carbon fixation linearly to transpiration), or from the Penman-Monteith model, which computes evapotranspiration based on weather conditions (Penman, 1948; Monteith, 1964). When transpiration is calculated based on a water use efficiency parameter, the user needs to provide evaporation values; when the Penman-Monteith model is used, transpiration and evaporation are separated by OpenSimRoot solving the Penman-Monteith model twice, once for full crop cover, and once for a bare soil. Based on the percent light capture by the crop OpenSimRoot scales evaporation and the transpiration terms assuming evaporation is negligible and small under full crop cover (Leaf Area Index ~3).

To simulate the soil hydrology, OpenSimRoot has a submodule that solves the Richards equation in three dimensions using finite element method (FEM) on a Cartesian grid. The soil water submodule is a simplified and modified C++ rewrite of the SWMS3D model, which is the basis of Hydrus and R-SWMS (Šimunek *et al.*, 1995; Diamantopoulos *et al.*, 2013).

Certain exceptional circumstances such as drainage or water ponding at top soil, are excluded. The top boundary condition is a water flux that is the difference between precipitation and evaporation. Evaporation, as computed by the Penman-Monteith equation, is assumed to be potential evaporation (i.e. appropriate for wet soils), and assumed to be equal across the soil surface, shoot geometry is not simulated. Potential evaporation is scaled back to an actual evaporation by including a smooth scaling function which causes evaporation to decrease smoothly from potential, when the top soil is wet, to equal the soil conductivity when the soil is not able to sustain higher evaporation rates. If the top soil is not necessarily uniformly wet, actual evaporation will be non-uniform across the soil surface in OpenSimRoot. The water retention curve and soil hydraulic conductivity are computed using the van Genuchten and Mualem equations.

The Richards equation can include a sink term, which in OpenSimRoot represents water uptake by roots (as described evaporation sink is handled as dynamic boundary condition). To do so we need to know 1) how much water is taken up by each root segment at a given moment in time, and 2) how that uptake is coupled to the FEM nodes of the grid on which the Richards equation is solved. Assuming that root uptake equals transpiration, i.e. we ignore temporal water storage in the plant, OpenSimRoot can either divide the water uptake of the whole root system by assuming each root segment contributes equally to uptake relative to its length (as in Hopmans, (Hopmans & Bristow, 2002)) or by solving the hyrdraulic architecture represented by a network model and using a circuit analogy likewise motivated by finite element theory (Alm *et al.*, 1992; Doussan *et al.*, 1998). The network model is novel in OpenSimRoot implemented to work with a growing root and used in the study of Schneider (Unpublished). This model requires axial and radial hydraulic conductivities for each root segment, which can be defined in the input files as a function of root age and class, and are scaled (i.e. normalized) with the inverse of the root segment length (axial), or the root segment surface area (radial). The coupling of the root model to the FEM model enables each root segment to have a soil water content at the root surface. The next step is to make sure that water uptake by the root system equals the transpiration which is

achieved by changing the water potential at the root collar (top of the hypocotyl). Getting the root collar potential is a parabolic optimization function which is solved with a newton solver, typically in three steps. The water potential at the top of the hypocotyl is not allowed to drop below a given threshold. If the threshold is reached, OpenSimRoot assumes that water uptake is less than potential transpiration and will write a warning. Further simulation results might not be correct as currently no effects of drought on photosynthesis, leaf expansion etc have been implemented. However, the model should correctly deal with compensatory uptake of water when soil water distribution is heterogeneous. And this model can show water loss of roots while the same conductivity from xylem to soil is assumed.
Mapping the root model to the FEM model is done based on a neighborhood search. All FEM nodes surrounding the root segment are considered. Sink terms, and local environment are computed based on inverse distance weighted average of the FEM nodes surrounding the root node. An alternative mapping algorithm, by which every FEM node is assigned with every root node has been implemented, in order to ignore root architecture completely in the water and nutrient uptake simulations. This was for example used in Postma and Lynch (2012) where it was concluded that the positioning of the root, that is root architecture, is necessary for simulating niche differentiation for nitrate uptake among maize, bean and squash plants, whereas if roots would be able to take up nutrients from everywhere in the soil, there would be no niche differentiation.

Nutrient module

OpenSimRoot has a nutrient module to simulate the uptake solutes, and in the new version theoretically simultaneously for various nutrients. This module was implemented to simulate the function of root architectural traits for nutrient uptake, and test tradeoffs for acquisition of different nutrients. Time dependent optimal and minimal nutrient content (μmol/g) have to be defined for leaves, stems and all root classes, for to be simulated solutes. These amounts are used to compute nutrient requirements of the plant, and compared to total uptake amounts, including initial seed reserves (for uptake see below). When uptake is less than demand, plant stress is assumed, with maximum stress being defined as uptake equal to minimal nutrient content (stress(uptake) = max(0, min((uptake-minimal)/(optimal-minimal),1)). Stress modifying impact functions can be defined for components such as leaf expansion rate, photosynthesis rates, respiration rates, and root elongation rates or secondary growth. Typically, they should be defined such that, when stress=0, growth ceases altogether. For example, by making the initial response of the shoot stronger than that of the root, the plant will decrease shoot to root ratios when nutrient deficient. Thus OpenSimRoot will move towards a functional equilibrium, although due to the inherent slow nature of growth, and the relative fast dynamics of other processes, this functional equilibrium might not be reached, and oscillatory behavior might occur (Postma & Lynch, 2011; Postma et al., 2014b). The current implementation assumes that internally, reallocation of nutrients is fast and perfect, such that all organs experience equal stress. This might be true for a nutrient like nitrogen, which typically causes chlorosis everywhere in the shoot, but might not be correct for other nutrients. The importance of simulation of nutrient redistribution in the plant still needs study, and would require implementation of a shoot architectural model in which the age and position of individual leafs is tracked.
Nutrient uptake from soil to root is simulated independently of utilization of nutrients within the plant. Two options for simulation are provided: 1) The Barber-Cushman model and 2) a 3D FEM

model. One is a C++ implementation of the original Barber-Cushman model with root hairs. The model is described as radial 1D PDE (Partial Differential Equation) which corresponds to the rhizosphere around the root. It assumes nutrient uptake to be described by a Michealis-Menten term, and the nutrient transport in the soil to be driven by convection (water flow) and diffusion. A buffer constant replaces a reaction term. The Barber-Cushman model is suitable for immobile nutrients like phosphorus. Phosphorus uptake causes steep gradients in concentrations around the root. These depletion zones are typically only 2-4 mm in diameter, and thereby would require a computationally unacceptably high resolution of the 3D finite element model (~0.1 mm resolution of a 1 m^3 soil pedon would result in $1e^{12}$ elements or 8 petabytes to hold a single double precision array).

Competition between roots is computed based on a local average root density which determines the outer boundary of the Barber-Cushman model. OpenSimRoot updates this boundary when new roots grow in the vicinity of other roots and corrects the initial nutrient concentration for new roots with the uptake of nutrients of older roots. Nevertheless, this handling of root competition is only acceptable when the overlap of depletion zones, which can be computed based on raster images of the root system, is relatively small. For crops, overlap in phosphorus depletion zones is typically below 20% because of its low mobility. Inter and intra root competition plays a much more important role in the uptake of mobile nutrients such as nitrate. Nitrate might form diffuse or no depletion zones around the root and for this reason is better simulated using a 3D FEM. SimRoot solved the convection-dispersion equation on the same FEM grid as the water transport is solved which can be restricting, OpenSimRoot alternatively can solve it on a refined grid, where the refinement factor is yet fixed to 2nd, 4th, 8th or the 16th of a reference grid. For each solute a new FEM model is instantiated and linked to the water model. The 3D FEM model for solute transport is coupled to the root systems using the same method as used for the hydraulic model, where the uptake of solutes by the root segments is based on Michaelis Menten kinetics, as in the Barber-Cushman model. Buffering and diffusion coefficients are dependent on the soil water content, and might thereby deviate from the constant coefficients used in the Barber-Cushman model. The effects must be considered when comparing the output of both models (Postma and Lynch, 2011).

When simulating more than one solute, solutes do not influence each other directly in OpenSimRoot. Indirect effects occur through the influence of nutrient uptake on root growth. Each solute has a stress function to determine how each impacts, for example, photosynthesis. A user specified aggregation function determines the aggregate impact (Dathe et al., 2013). For example, Postma et al., (2014a) showed how the optimal lateral branching density in maize depends on the relative availability of phosphorus and nitrogen.

References

Alm DM, Cavelier J, Nobel PS. **1992**. A finite-element model of radial and axial conductivities for individual roots: development and validation for two desert succulents. *Annals of Botany* **69**: 87–92.

Dathe A, Postma JA, Lynch JP. **2013**. Modeling resource interactions under multiple edaphic stresses. In: Timlin D,, In: Ahuja LR, eds. Advances in Agricultural Systems Modeling. Enhancing Understanding and Quantification of Soil–Root Growth Interactions. Madison, Wis., USA: American Society of Agronomy, Crop Science Society of America, Soil Science Society of America., 273–294.

Diamantopoulos E, Iden SC, Durner W. **2013**. Modeling non-equilibrium water flow in multistep outflow and multistep flux experiments. *HYDRUS Software Applications to Subsurface Flow and Contaminant Transport Problems*: 69.

Doussan C, Pagès L, Vercambre G. **1998**. Modelling of the hydraulic architecture of root systems: An integrated approach to water absorption - Model description. *Annals of Botany* **81**: 213–223.

Hopmans JW, Bristow KL. **2002**. Current capabilities and future needs of root water and nutrient uptake modeling. *Advances in Agronomy* **77**: 103–183.

Monteith JL. **1964**. Evaporation and environment. *Symposia of the society for experimental biology* **19**: 205–234.

Penman HL. **1948**. Natural evaporation from open water, bare soil and grass. Proceedings of the Royal Society of London A: Mathematical, Physical and Engineering Sciences. The Royal Society, 120–145.

Postma JA, Dathe A, Lynch JP. **2014a**. The optimal lateral root branching density for maize depends on nitrogen and phosphorus availability. *Plant Physiology* **166**: 590–602.

Postma JA, Lynch JP. **2011**. Theoretical evidence for the functional benefit of root cortical aerenchyma in soils with low phosphorus availability. *Annals of Botany* **107**: 829–841.

Postma JA, Lynch JP. **2012**. Complementarity in root architecture for nutrient uptake in ancient maize/bean and maize/bean/squash polycultures. *Annals of Botany* **110**: 521–534.

Postma JA, Schurr U, Fiorani F. **2014b**. Dynamic root growth and architecture responses to limiting nutrient availability: linking physiological models and experimentation. *Biotechnology Advances* **32**: 53–65.

Šimunek J, Huang K, van Genuchten MT. **1995**. *The SWMS 3D code for simulating water flow and solute transport in three-dimensional variably-saturated media*. California: U. S. Salinity laboratory, USDA.

Note S6: Example of a simple OpenSimRoot input file

The XML below is an example of an OpenSimRoot input file that constructs a simple crop model, without any roots. All the SimulaX tags will instantiate a minimodel of the corresponding type, for example a constant (time independent parameter) is declared as <SimulaConstant ...>. Metadata for the minimodels, such as name and unit, are given in the attributes lists.

General rules for XML documents

1) The document has tags which are between brackets like <>
2) Tags correspond to minimodels in OpenSimRoot and therefore carry different names, such as SimulaBase, SimulaConstant, etc.
3) Tags need to be closed either by putting a / before the closing bracket, or if data is nested inside the tag with a corresponding closing tag which is recognized by </. For example <SimulaConstant></SimulaConstant>
4) Between opening and closing tags you will find data, and or declarations of minimodels which are at the next level in the hierarchy
5) Tags carry attributes which describe metadata. Attributes are always listed as attribute=”something”. In OpenSimRoot all tags have at least a name attribute.
6) An XML document is plain text and recognized by a special declaration at the top of the document. <?**xml** version="1.0" encoding="UTF-8"?>
7) XML documents can have stylesheets associated with them so the the browser knows how to render the document. Here we have <?xml-stylesheet type="text/xsl" href="tree-view.xsl"?>
8) Comments are between <!-- and -->.
9) All XML documents of a document type tag. For OpenSimRoot the document type is declared as <SimulationModel></SimulationModel>. All other tags must be in between these tags.

Here follows an example input file. The comments in black give more explanation as to how a simple crop model is being constructed by this input file. Input files for full root architectural models can be found in the software repository on gitlab:
https://gitlab.com/rootmodels/OpenSimRoot

```
<?xml version="1.0" encoding="UTF-8"?>
<?xml-stylesheet type="text/xsl" href="tree-view.xsl"?>
<!--
Copyright © 2016 Forschungszentrum Jülich GmbH
All rights reserved.

Redistribution and use in source and binary forms, with or without modification, are permitted
under the GNU General Public License v3 and provided that the following conditions are met:
1. Redistributions of source code must retain the above copyright notice, this list of conditions
and the following disclaimer.
2. Redistributions in binary form must reproduce the above copyright notice, this list of
conditions and the following disclaimer in the documentation and/or other materials provided with
the distribution.
3. Neither the name of the copyright holder nor the names of its contributors may be used to
endorse or promote products derived from this software without specific prior written permission.

Disclaimer
THIS SOFTWARE IS PROVIDED BY THE COPYRIGHT HOLDERS AND CONTRIBUTORS "AS IS" AND ANY EXPRESS OR
IMPLIED WARRANTIES, INCLUDING, BUT NOT LIMITED TO, THE IMPLIED WARRANTIES OF MERCHANTABILITY AND
FITNESS FOR A PARTICULAR PURPOSE ARE DISCLAIMED. IN NO EVENT SHALL THE COPYRIGHT HOLDER OR
CONTRIBUTORS BE LIABLE FOR ANY DIRECT, INDIRECT, INCIDENTAL, SPECIAL, EXEMPLARY, OR CONSEQUENTIAL
DAMAGES (INCLUDING, BUT NOT LIMITED TO, PROCUREMENT OF SUBSTITUTE GOODS OR SERVICES; LOSS OF USE,
```

```
DATA, OR PROFITS; OR BUSINESS INTERRUPTION) HOWEVER CAUSED AND ON ANY THEORY OF LIABILITY,
WHETHER IN CONTRACT, STRICT LIABILITY, OR TORT (INCLUDING NEGLIGENCE OR OTHERWISE) ARISING IN ANY
WAY OUT OF THE USE OF THIS SOFTWARE, EVEN IF ADVISED OF THE POSSIBILITY OF SUCH DAMAGE.

You should have received the GNU GENERAL PUBLIC LICENSE v3 with this file in license.txt but it
can also be found at http://www.gnu.org/licenses/gpl-3.0.en.html -->

<!--This XML constructs a simple, radiation use efficiency based crop model.

Roots and stems are only presented as Carbon (dry weight) pools
Leaf dry weight is converted to leaf area based on specific leaf area (SLA)
Leaf area is converted to light interception using an extinction coefficient.
Light interception is converted to photosynthesis using radiation use efficiency (RUE).
Photosynthesis is converted to structural carbon using a conversion factor (multiplier) which
represents relative losses due to respiration
Fixed allocation causes structural carbon to be divided over root, stem and leafs.

Behavior, is simple exponential growth for which
RGR = SLA * C2Leafs * photosynthesis * multiplier
However, as the light interception with increasing leaf area
reaches an asymptote, the model will move towards linear growth.-->

<SimulationModel>

<!-- SimulaBase is a simple container, that holds other SimulaX objects. SimulaBase is thus a
minimodel that does not hold or simulate data. It should, like all mini models, have a name. So
here we declare a container in which we are going to put all our plants. Inside it we put a
container for our plant, named arbitrarily “myPlant”.   -->

    <SimulaBase name="plants">
      <SimulaBase name="myplant">

<!-- Here follow three SimulaConstant declarations. SimulaConstant is a minimodel that holds time
and space independent data of different types. Possible types are double, int, string,
Coordinate. Besides the name attribute they must have a unit, and if the data is not a double, a
type declaration.

A plant should be of a given species/genotype. The model will look for a parameter set in
roottypeParameters with the corresponding type. Here we declare that we want to simulate a plant
of type mySpecies -->

        <SimulaConstant name="plantType" type="string">
            mySpecies
        </SimulaConstant>

<!-- The time that the plant is planted. 0. is at the start of the simulation. -->

        <SimulaConstant name="plantingTime" unit="day">
            0.
        </SimulaConstant>

<!-- Location in space where the seed is planted.  -->

        <SimulaConstant name="plantPosition" type="Coordinate">
            0 -2 0

<!-- Container that hold all the minimodels that will simulate shoot
related parameters. The shoot and root are inside plantPosition, as OpenSimRoot works with a
relative Coordinate system. We achieve that all coordinates that belong to our plant are relative
to plantPosition. -->

            <SimulaBase name="shoot">

<!-- Licht interception is simulated by the light interception module. SimulaDerivative declares
a minimodel that wil use the lightInterception plugin to compute light interception. Attributes
are name of what is being computed (name="lightInterception"), the unit of what is being computed
(unit="umol/cm2/day"), and the plugin that should be used to compute it
(function="lightInterception"). The plugin lightInterception requires leafAreaIndex and from the
parameter section and extinctionCoefficient (kdf). Further it needs irradiation levels from the
environmental section. All have been declared further down.  -->
```

```
              <SimulaDerivative name="lightInterception" unit="umol/cm2/day"
                function="lightInterception" />

<!--Simulation of photosynthesis rates can be done by the plugin registered as
photosynthesisLintulV2. However, since we want to know the total photosynthesis, the rates need
to be integrated over time. SimulaVariable does this. Thus unit is not g/day, but g. Attributes
are otherwise same as for a SimulaDerivative tag. Optional attributes that control the method of
integration and the timestep can be given. For example integrationFunction="ForwardEuler" will
use the forward euler plugin for integrating. List of all integration methods can be obtained by
running OpenSimRoot -L. maximumTimeStep="0.1" would reduce the maximum timestep from the default
0.2 to 0.1.
-->
              <SimulaVariable name="photosynthesis" unit="g"
                function="photosynthesisLintulV2" />

<!--Declaration of how leafAreaIndex should be simulated, as it is needed by the
lightInterception plugin. -->

              <SimulaDerivative name="leafAreaIndex" unit="cm2/cm2"
                function="leafAreaIndex" />

<!--Declaration of how leafArea should be simulated, as it is needed by the leafAreaIndex plugin.
Here the initial leaf area is given. More time value pairs can be entered in order to specify a
predefined initial leaf area. The leafArea plugin will simulate increases in leaf area on the
basis of carbon allocation to the leafs, the specificLeafArea and the carbonToDryweight ratio,
all declared later on.--->

              <SimulaVariable name="leafArea" unit="cm2" function="leafArea">
                0. 1. </SimulaVariable>

<!--Same as leafArea, but then for leafDryWeight. -->

              <SimulaVariable name="leafDryWeight" unit="g"
                function="leafDryWeight.v2"> 0. 0.001 </SimulaVariable>

<!--Here follow more minimodels, all with their respective plugins declared -->

              <SimulaDerivative name="relativeCarbonAllocation2Leafs"
                unit="100%"
                function="relativeCarbonAllocation2LeafsFromInputFile" />
              <SimulaVariable name="carbonAllocation2Leafs" unit="g"
                function="carbonAllocation2Leafs" />
              <!-- optional to have stem weight -->
              <SimulaDerivative name="relativeCarbonAllocation2Stems"
                unit="100%"
                function="relativeCarbonAllocation2StemsOneMinusLeafs" />
              <SimulaVariable name="carbonAllocation2Stems" unit="g"
               function="carbonAllocation2Stems" />
              <SimulaVariable name="stemDryWeight" unit="g"
               function="stemDryWeight" />
            </SimulaBase>
        </SimulaConstant>

<!--In this simulation it was decided to declare the carbonToDryWeight ratio as a simple
constant. -->

        <SimulaConstant name="carbonToDryWeightRatio" unit="100%">
            0.45
        </SimulaConstant>

<!--Carbon allocation -->

        <SimulaDerivative name="relativeCarbonAllocation2Shoot"
            unit="100%"
            function="relativeCarbonAllocation2ShootFromInputFile" />
        <SimulaVariable name="carbonAllocation2Shoot" unit="g"
            function="carbonAllocation2Shoot" />
```

```
<!--Instead of using a process specific plugin to simulate the carbon available for growth, here
we use a general plugin named usePath which simply couples the carbon available for growth to
photosynthesis. Since this declaration as a child called “multiplier” the photosynthesis rates is
halved, so it is assumed that half of all carbon fixed by photosynthesis is converted to plant
dry mass, the rest is respired. -->

        <SimulaDerivative name="carbonAvailableForGrowth"
            unit="g" function="usePath">
            <SimulaConstant name="path" type="string">
              plantPosition/shoot/photosynthesis
            </SimulaConstant>
            <!-- half of carbon assumed to be respired -->
            <SimulaConstant name="multiplier">0.5</SimulaConstant>
        </SimulaDerivative>

<!--Some declarations related to roots -->

      <SimulaDerivative name="relativeCarbonAllocation2Roots"  unit="100%"
               function="relativeCarbonAllocation2RootsOneMinusShoot" />
      <SimulaVariable name="carbonAllocation2Roots" unit="g"
               function="carbonAllocation2Roots" />
      <SimulaVariable name="rootDryWeight" unit="g" function="rootDryWeight" />

<!--The closing tags for the myPlant and Plants containers. -->

      </SimulaBase>
    </SimulaBase>



<!-- Environmental data needs to be declared, here all we need is irradiation in order to know
how much light is being captured for photosynthesis -->

    <SimulaBase name="environment">
      <SimulaBase name="atmosphere">
        <SimulaTable name_column1="time" name_column2="irradiation"
            unit_column1="day" unit_column2="umol/cm2/day">
            0 3000
            100 3000
        </SimulaTable>
      </SimulaBase>
    </SimulaBase>

<!-- here a parameter section for our plant is specified. -->

    <SimulaBase name="rootTypeParameters">
      <SimulaBase name="mySpecies">
        <SimulaBase name="resources">

<!--relativeCarbonAllocation to leafs (see above) uses a plugin in that simply looks up data from
a table. The table is declared here. SimulaTables have two columns. Each column has a name and a
unit declared in the attribute list. Here, as will be often the case, the first column is time.
This is time since the plant started growing, not since the start of the simulation. First all
carbon that is going to the shoot is allocated to leafs, later on more carbon is going to the
stems. Values in the table are interpolated linearly, unless a different interpolation method is
declared. Currently, only interpolation=”step” is implemented as alternative method.  -->

            <SimulaTable name_colum1="time" unit_colum1="day"
              name_colum2="carbonAllocation2LeafsFactor" unit_colum2="100%">
              0 1
              10 0.8
              40 0.5
              60 0.
              80 0.
            </SimulaTable>

<!--How much carbon should go to the root. The rest goes to the shoot. -->

            <SimulaTable name_colum1="time" unit_colum1="day"
              name_colum2="carbonAllocation2RootsFactor" unit_colum2="100%">
```

```
              0 0.8
              10 0.2
              40 0.2
              80 0.2
            </SimulaTable>
        </SimulaBase>

<!--Declaration of several well known shoot related parameters. -->

        <SimulaBase name="shoot">
            <SimulaConstant name="areaPerPlant" unit="cm2">
              100
            </SimulaConstant>
            <SimulaConstant name="extinctionCoefficient" unit="noUnit">
              0.6
            </SimulaConstant>
            <SimulaConstant name="lightUseEfficiency" unit="g/umol">
              0.4E-6
            </SimulaConstant>
            <SimulaTable name_colum1="time" name_colum2="specificLeafArea"
              unit_colum1="day" unit_colum2="g/cm2" note="SLA in lintul">
              0 0.001
              10 0.002
              40 0.003
              80 0.003
            </SimulaTable>
        </SimulaBase>
      </SimulaBase>
    </SimulaBase>

<!--This section gives the user some control over the output.-->

    <SimulaBase name="simulationControls">
      <SimulaBase name="outputParameters">
        <SimulaBase name="table">

<!--A table should be written containing values for each minimodel, for every half day from day 0
to 80. Hierarchy will be traversed up to depth 10 -->

            <SimulaConstant name="run" type="bool"> 1 </SimulaConstant>
            <SimulaConstant name="searchingDepth" type="int"> 10
            </SimulaConstant>
            <SimulaConstant name="startTime" type="time"> 0.
            </SimulaConstant>
            <SimulaConstant name="endTime" type="time"> 80.
            </SimulaConstant>
            <SimulaConstant name="timeInterval" type="time"> 0.5
            </SimulaConstant>
        </SimulaBase>
      </SimulaBase>
    </SimulaBase>

<!--We are done -->

</SimulationModel>
```

User friendly viewing of XML input files

A webbrowser can transform this into more human friendly presentation using the attached tree-view.xsl transformation style sheet (available for download at the gitlab repository https://gitlab.com/rootmodels/OpenSimRoot). The result when you open this file in a browser is given below.

OpenSimRoot Parametrization
OpenSimRoot uses a hierarchical xml formatted input file which is graphically presented below. The hierarchy gives the parameters context. For example, the parameter 'specific leaf area' belongs to the shoot of a specific plant. In OpenSimRoot parameters can be a single value, a value drawn from a distribution, or the result of an interpolation table.

```
|___ Origin
     |___ 'plant'
     |    |___ 'myplant'
     |         |___ 'plantType' = mySpecies
     |         |___ 'plantingTime' = 0 (day)
     |         |___ 'plantPosition' = 0 -2 0
     |         |    |___ 'shoot'
     |         |         |___ 'lightInterception' (umol/cm2/day)
     |         |         |___ 'photosynthesis' (g)
     |         |         |___ 'leafAreaIndex' (cm2/cm2)
     |         |         |___ 'leafArea' (cm2) initial value = 1.
     |         |         |___ 'leafDryWeight' (g)
     |         |         |___ 'relativeCarbonAllocation2Leafs' (100%)
     |         |         |___ 'carbonAllocation2Leafs' (g)
     |         |         |___ 'relativeCarbonAllocation2Stems' (100%)
     |         |         |___ 'carbonAllocation2Stems' (g)
     |         |         |___ 'stemDryWeight' (g)
     |         |___ 'carbonToDryWeightRatio' = 0.45 (100%)
     |         |___ 'relativeCarbonAllocation2Shoot' (100%)
     |         |___ 'carbonAllocation2Shoot' (g)
     |         |___ 'carbonAvailableForGrowth' (g)
     |         |    |___ 'path' = plantPosition/shoot/photosynthesis
     |         |    |___ 'multiplier' = 0.5
     |         |___ 'relativeCarbonAllocation2Roots' (100%)
     |         |___ 'carbonAllocation2Roots' (g)
     |         |___ 'rootDryWeight' (g)
     |___ 'environment'
     |    |___ 'atmosphere'
     |         |___ x,y pairs :{ 0 3000 100 3000 }
     |___ 'rootTypeParameters'
     |    |___ 'mySpecies'
     |         |___ 'resources'
     |         |    |___ 'carbonAllocation2LeafsFactor' (100%)=f{'time'} (day) x,y pairs :{ 0 1 10 0.8 40 0.5 60 0. 80 0. }
     |         |    |___ 'carbonAllocation2RootsFactor' (100%)=f{'time'} (day) x,y pairs :{ 0 0.8 10 0.2 40 0.2 80 0.2 }
     |         |___ 'shoot'
     |              |___ 'areaPerPlant' = 100 (cm2)
     |              |___ 'extinctionCoefficient' = 0.6 (noUnit)
     |              |___ 'lightUseEfficiency' = 0.4E-6 (g/umol)
     |              |___ 'specificLeafArea' (g/cm2)=f{'time'} (day) x,y pairs :{ 0 0.001 10 0.002 40 0.003 80 0.003 }
     |___ 'simulationControls'
          |___ 'outputParameters'
               |___ 'table'
                    |___ 'run' = 1
                    |___ 'searchingDepth' = 10
                    |___ 'startTime' = 0.
                    |___ 'endTime' = 80.
                    |___ 'timeInterval' = 0.5
```

User friendly editing of XML input files

Besides attaching a transformation sheet for transforming xml to html and view it in a webbrowser, a XML schema (xsd) is available, which allows schema aware editors to provide auto completion and validation of the input file. Below is a screenshot from an XML editor (plugin in www.eclipse.org) which shows the declaration of the schema, and a pop down menu for the available arguments for SimulaTable, and the different values that the objectGenerator argument can have.

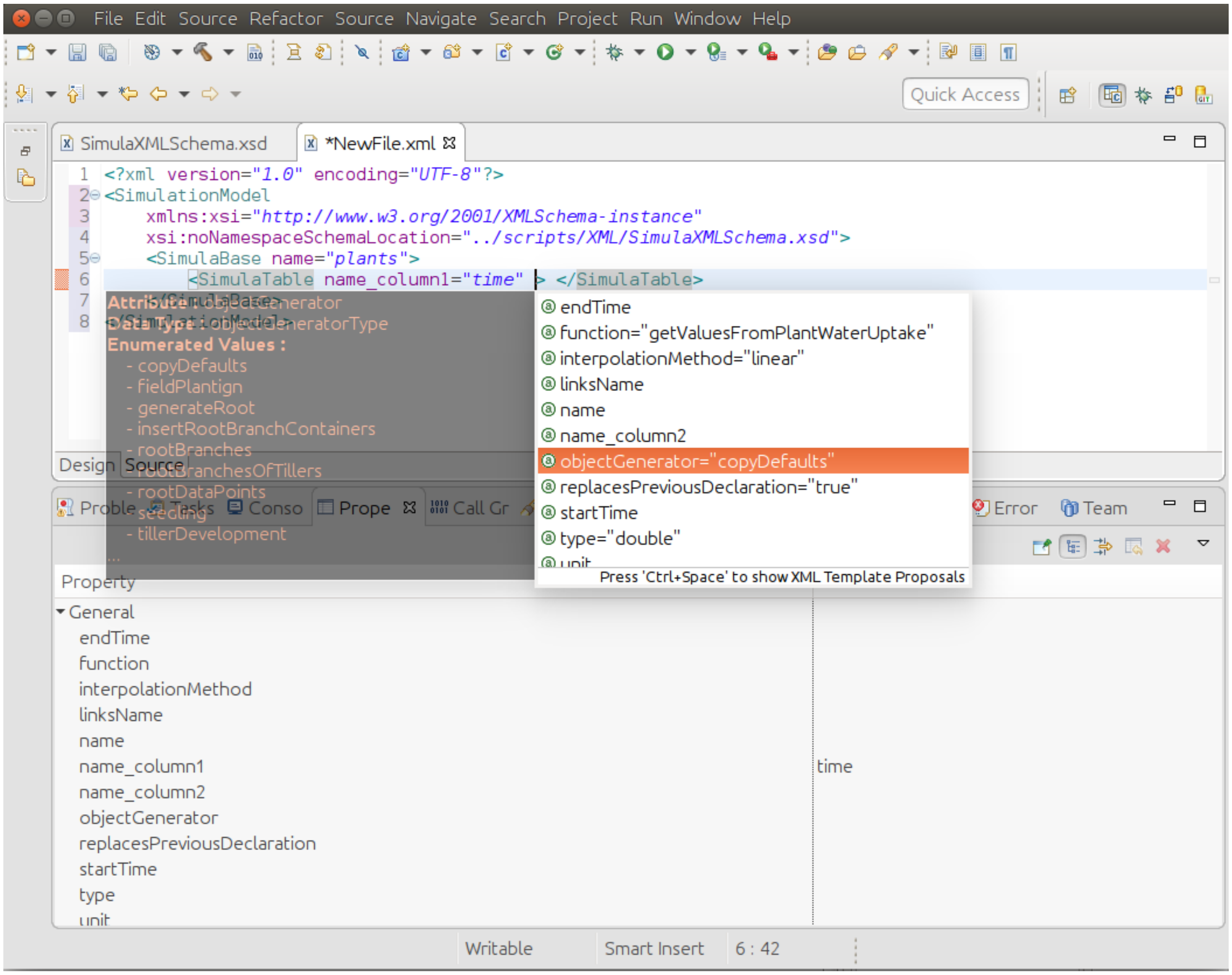

Figure S6.1: Screenshot of XML editor in eclipse in which a new file was created, using the new file wizard. The schema is declared with "`xsi:noNamespaceSchemaLocation="../scripts/XML/SimulaXMLSchema.xsd"`" and the black and the black and white pop up boxes show suggestions, as defined in the schema.

Note S7: Diagram of all state variables and their dependencies in an exemplar bean simulation

We drew a graph which contains the various state variables in an example simulation and the dependencies among them. Each state variable is simulated by a SimulaObject, here we depicted SimulaConstants, SimulaTables and SimulaStochastic as wedges, whereas all others are depicted as a rounded boxes. The arrows indicate information flow, that is the result of one minimodel goes into the computation of another. The network is strongly dependent on the input file, and somewhat dependent on time, given that computations might switch on given conditions and should thereby be regarded as exemplar. To properly view the graph, enlarge the pdf strongly.

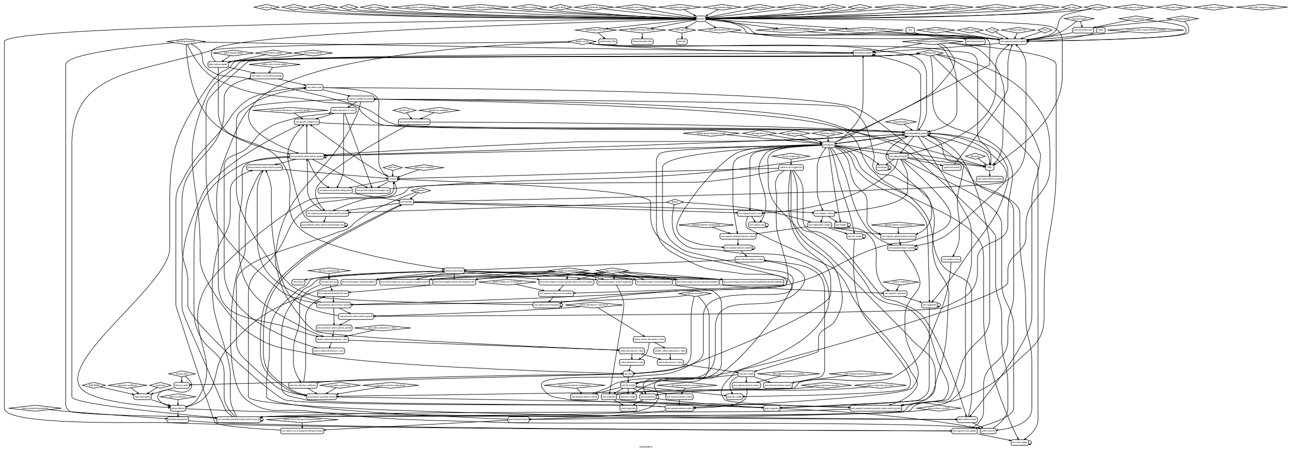

Figure S7.1: Graph representing all the state variables in a bean simulation, and their connections at day 12. For better viewing, enlarge by about 1200%.
